# The linear mitochondrial genome of the quarantine chytrid *Synchytrium endobioticum*; insights into the evolution and recent history of an obligate biotrophic plant pathogen

**DOI:** 10.1186/s12862-018-1246-6

**Published:** 2018-09-10

**Authors:** Bart T. L. H. van de Vossenberg, Balázs Brankovics, Hai D. T. Nguyen, Marga P. E. van Gent-Pelzer, Donna Smith, Kasia Dadej, Jarosław Przetakiewicz, Jan F. Kreuze, Margriet Boerma, Gerard C. M. van Leeuwen, C. André Lévesque, Theo A. J. van der Lee

**Affiliations:** 1 Wageningen UR, Droevendaalsesteeg 1, Biointeractions and Plant Health & Plant Breeding, 6708 PB Wageningen, The Netherlands; 2 Dutch National Plant Protection Organization, National Reference Centre, Geertjesweg 15, 6706EA Wageningen, The Netherlands; 3 Westerdijk Fungal Biodiversity Institute, Uppsalalaan 8, 3584 Utrecht, CT Netherlands; 4 Agriculture and Agri-Food Canada, 960 Carling Avenue, Ottawa, Canada; 5 Canadian Food Inspection Agency, 93 Mount Edward Road, Charlottetown, Canada; 6 Plant Breeding and Acclimatization Institute, National Research Institute, 05-870 Blonie, Radzikow, Warsaw, Poland; 7 International Potato Centre, Avenida La Molina, 1895 Lima, Peru; 8 Hilbrands Laboratorium BV, Kampsweg 27, 9418 PD Wijster, Wijster, The Netherlands

**Keywords:** Mitochondrial haplotypes, Pest introduction, Population dynamics, Fungal communities, Pathotype formation, Chytridiomycota

## Abstract

**Background:**

Chytridiomycota species (chytrids) belong to a basal lineage in the fungal kingdom. Inhabiting terrestrial and aquatic environments, most are free-living saprophytes but several species cause important diseases: e.g. *Batrachochytrium dendrobatidis*, responsible for worldwide amphibian decline; and *Synchytrium endobioticum*, causing potato wart disease. *S. endobioticum* has an obligate biotrophic lifestyle and isolates can be further characterized as pathotypes based on their virulence on a differential set of potato cultivars. Quarantine measures have been implemented globally to control the disease and prevent its spread. We used a comparative approach using chytrid mitogenomes to determine taxonomical relationships and to gain insights into the evolution and recent history of introductions of this plant pathogen.

**Results:**

We assembled and annotated the complete mitochondrial genome of 30 *S. endobioticum* isolates and generated mitochondrial genomes for five additional chytrid species. The mitochondrial genome of *S. endobioticum* is linear with terminal inverted repeats which was validated by tailing and PCR amplifying the telomeric ends. Surprisingly, no conservation in organisation and orientation of mitochondrial genes was observed among the Chytridiomycota except for *S. endobioticum* and its sister species *Synchytrium microbalum*. However, the mitochondrial genome of *S. microbalum* is circular and comprises only a third of the 72.9 Kbp found for *S. endobioticum* suggesting recent linearization and expansion. Four mitochondrial lineages were identified in the *S. endobioticum* mitochondrial genomes. Several pathotypes occur in different lineages, suggesting that these have emerged independently. In addition, variations for polymorphic sites in the mitochondrial genome of individual isolates were observed demonstrating that *S. endobioticum* isolates represent a community of different genotypes. Such communities were shown to be complex and stable over time, but we also demonstrate that the use of semi-resistant potato cultivars triggers a rapid shift in the mitochondrial haplotype associated with increased virulence.

**Conclusions:**

Mitochondrial genomic variation shows that *S. endobioticum* has been introduced into Europe multiple times, that several pathotypes emerged multiple times, and that isolates represent communities of different genotypes. Our study represents the most comprehensive dataset of chytrid mitogenomes, which provides new insights into the extraordinary dynamics and evolution of mitochondrial genomes involving linearization, expansion and reshuffling.

**Electronic supplementary material:**

The online version of this article (10.1186/s12862-018-1246-6) contains supplementary material, which is available to authorized users.

## Background

*Synchytrium endobioticum* (Schilb.) Perc. is a chytrid fungus (Chytridiomycota) causing potato wart disease, which is one of the most important quarantine diseases on cultivated potato [1, 2]. Host tissues infected with this fungus develop tumour-like galls (i.e. warts) rendering the crop unmarketable. The malformed tissue produces resting spores which are released into the surrounding soil when the host tissue decays, and once released, spores can remain viable and infectious for decades [3, 4]. Individual isolates can be further characterized as pathotypes based on their virulence on a differential set of potato cultivars. Reported from many countries worldwide, *S. endobioticum* is believed to originate from potato-growing areas in the Andean region from where it was taken into the United Kingdom in the aftermath of the Irish potato famine (~1880s) [5]*.* Initially, the disease spread quickly and widely in Europe, but phytosanitary measures restricted its movement to other parts of the world [2, 5]. Today, quarantine measures have been implemented through phytosanitary legislation world-wide. In addition, *S. endobioticum* is on the federal select agent program of the United States of America [6]. Restricting potato cultivation in infested fields and the use of resistant potato cultivars are the only known efficient control strategies. Characterization of potato wart resistance in potato cultivars is essential for efficient control.

Forty-five years after the first description of *S. endobioticum* [7], only a single pathotype was known, which is nowadays known as pathotype 1(D1). In 1941, wart development was discovered on formerly resistant potato cultivars (Edda, Edelragis, Parnassia, Primula, Sabina, and Sickingen) in Gießübel, Germany [8] and it was recognized that a new pathotype, pathotype 2(G1), of the fungus had emerged. Subsequently, new pathotypes were found in Germany, the Czech Republic, and Ukraine [9], and at present, 39 pathotypes are described with the most recent one being discovered in Piekielnik, Poland [10]. Although pathotype identity is essential for authorities to enforce suitable phytosanitary measures, these tests do not provide information on the migration and spread of the fungus, or the emergence of new pathotypes.

To infer intraspecific genetic variation, Gagnon et al. [11] developed polymorphic microsatellite loci for *S. endobioticum*. However, isolates of the same pathotype were found to display highly variable genotypes and none of the markers correlated with a specific pathotype. Apart from several Canadian isolates, no clustering based on geographical origin was observed. We have pursued a different approach, exploiting the mitochondrial genome to determine taxonomical relationships, and to gain insights into the introductions and population dynamics of this plant pathogen.

The mitochondrion is a cell organelle involved in ATP production, and is descended from an α-proteobacterium [12]. They have maintained the double membrane characteristic of their ancestors and retained a highly reduced bacterial-like genome [13, 14]. The mitochondrial genome is typically assumed to be a single maternally inherited circular molecule that is present in high copy number, ranging from a few hundred to a few thousand copies per cell. Loci from the mitochondria are most frequently used in phylogenetic and population-level studies [15–17], because of their copy number, commonly available generic primers for amplification and sequencing, and phylogenetic signal on higher taxonomical levels [18, 19]. With the introduction of next generation sequencing technologies, the number of complete mitochondrial genomes has rapidly increased [14]. However, complete mitochondrial genomes of the early diverging fungal lineages of the Chytridiomycota and Blastocladiomycota are scarce with only eight publically available complete and annotated mitochondrial genomes. This limits the possibilities for species level analyses using the mitochondrial genome in these divisions.

The aims for this study were to: (i) sequence, assemble and annotate the complete mitochondrial genome of *S. endobioticum*; (ii) perform phylogenetic analyses using the mitochondrial genes to determine its taxonomical relationships to 14 other species from the Chytridiomycota including *Synchytrium taraxaci*, which is the type species for the genus [20], and *Synchytrium microbalum*, the first culturable *Synchytrium* species to be described [21]; and (iii) perform network analyses using a panel of *S. endobioticum* isolates from different countries covering several pathotypes and identify potential linkages between genotypes, geographical origins and pathotypes.

## Results

### Assembly of the *S. endobioticum* mitochondrial genome

**Fig. 1:**
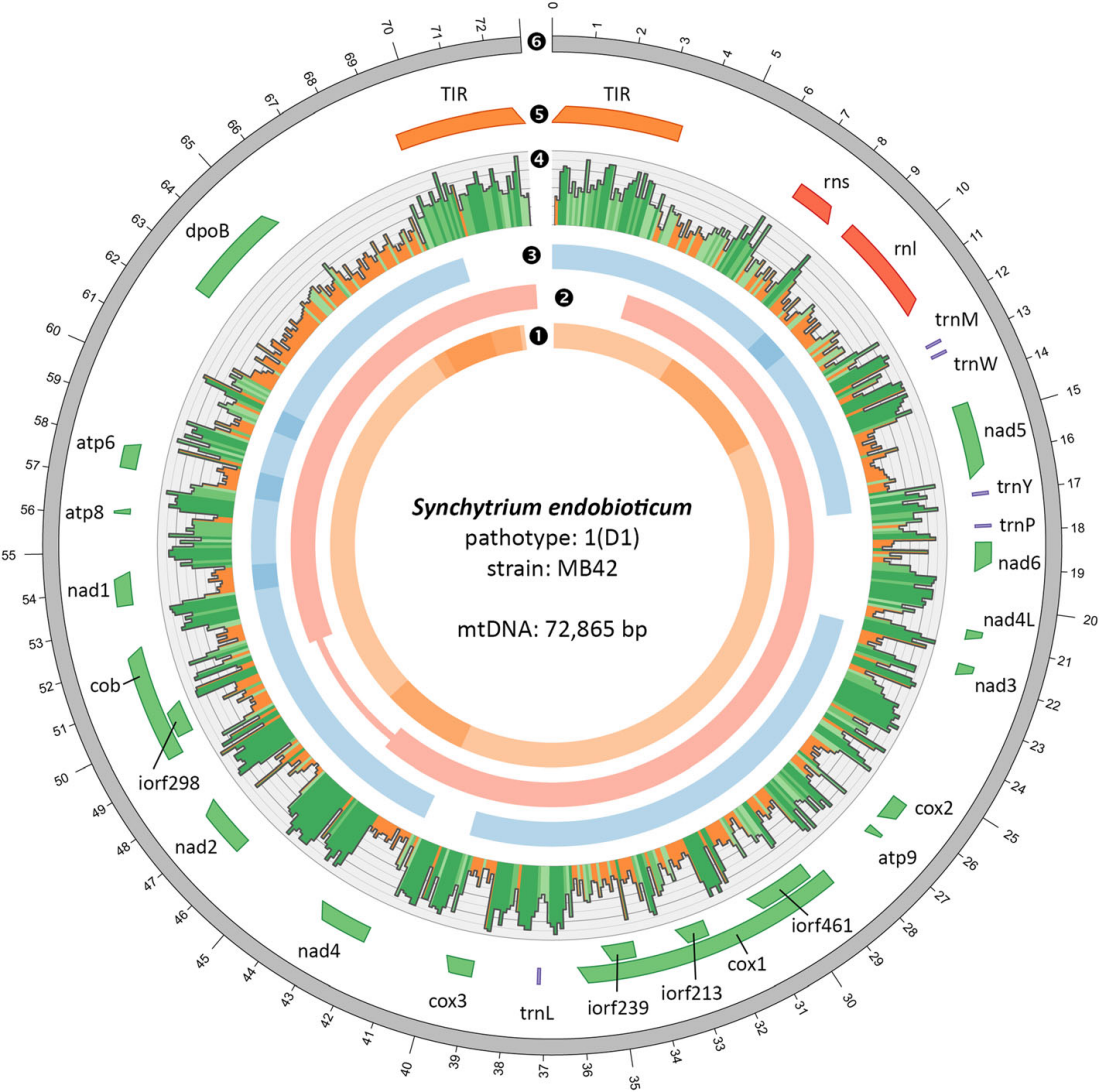
Assembly and annotation of the linear *S. endobioticum* mtDNA genome. Tracks ❶ to ❸ represent putative mtDNA scaffolds from three different assemblies (respectively putative mtDNA scaffolds from the *S. endobioticum* MB42 genome assembly, reference guided assembly with GRAbB using *S. endobioticum* MB42 HiSeq data, and a reference guided assembly using *S. endobioticum* MB42 PacBio CCS) mapped to the linear mtDNA genome. Darker shades are used to indicate regions where scaffolds from the same assembly overlap. The narrow line in track 2 indicates a gap relative to the linear mtDNA genome. ❹ G + C content determined with a 100 bp sliding window in which orange ≤40% G + C, light green 40–50% G + C, green 50–60% G + C, and dark green > 60% G + C. ❺ mtDNA annotation track showing genes (green), rRNA (red), tRNA (purple) and the terminal inverted repeats (orange). All genes, rRNAs and tRNAs are orientated in the 5′ to 3’direction. ❻ the 72,865 bp linear mitochondrial genome of *S. endobioticum*

We assembled and annotated the mitochondrial genome of *S. endobioticum* pathotype 1(D1) isolate MB42. Three independent assemblies using different datasets were performed and the results were combined to obtain a consensus mitochondrial genome; (i) from the MB42 reference genome, seven putative mitochondrial scaffolds were identified representing a total size of 68,068 base pairs (bp); (ii) using GRAbB, a single 63,627 bp putative mitochondrial scaffold was obtained; (iii) an assembly of PacBio data resulted in five putative mitochondrial scaffolds with a total size of 90,248 bp. Putative mitochondrial scaffolds were combined to create a single 72,865 bp consensus assembly. The assembly was verified by mapping the long-read PacBio data to the consensus sequence which resulted in contiguous sequence with no reads that exceed the 5′ or 3′ terminal ends (Additional file 1: Figure S1). No circular mapping was obtained for the linear mtDNA, i.e. no reads were found that spanned the terminal ends of the consensus sequence, and terminal deoxynucleotidyl transferase (TdT) tailing followed by PCR amplification further verified the linear ends of the molecule. The ends of the linear molecule contain two identical 3529 bp repeats in inverted orientation, which we refer to as Terminal Inverted Repeats (TIR) (Fig. 1, Additional file 1: Figure S2).

The annotation of the *S. endobioticum* mitochondrial genome recovered the mitochondrial genes typically found in fungal species: *atp6, atp8, atp9, cob, cox1, cox2, cox3, nad1, nad2, nad3, nad4, nad4L, nad5, nad6, rns* and *rnl*. In addition, a reduced set of five tRNA recognising methionine, tryptophan, tyrosine, proline and leucine was found. Four homing endonuclease genes (HEGs) were identified encoded by introns of protein coding genes: three HEGs residing in *cox1* introns carry the LAGLIDADG_1 Pfam domain (PF00961), whereas the HEG residing in the *cob* intron carries the LAGLIDADG_2 Pfam domain (PF03161). No presence/absence variations were found for the intron and HEGs content in other *S. endobioticum* isolates. Interestingly a gene coding for DNA polymerase type B (*dpoB*) containing the DNA_pol_B_2 Pfam domain (PF03175), which are also found in linear mitochondrial plasmids, was identified on the *S. endobioticum* mitogenome. The *dpoB* gene is partially repeated in the A + T rich region (Additional file 1: Figure S3).

Intergenic regions are found to have an unusual high G + C content (57.3%), with several intergenic spacers having G + C contents close to 70% (e.g. *nad4*-*nad2*: 68.5%, *nad2*-*cob*: 67.6%, and *cox3*-*nad4*: 65.5%). In contrast, the intergenic regions in the mitochondrial genome of a closely related species, *S. microbalum,* consist of 28.6% G + C, with the highest G + C percentage found in the *nad6*-*nad4L* intergenic spacer (34.2%). *S. endobioticum* has the highest intergenic G + C content in chytrid mitogenomes reported to date, which is only almost matched by the obligate biotrophic *S. taraxaci* (51.4%). For Chytridiomycota other than *S. endobioticum* and *S. taraxaci*, a mean intergenic G + C content of 34.3% (standard deviation 9.7%) was observed.

Of the five additional chytrid species sequenced in this study, three resulted in a single circular mtDNA scaffold containing all 14 mtDNA genes, i.e. *C. confervae* (225.6 kb), *S. palustris* (126.5 kb), and *S. microbalum* (23.8 kb). The assembly of *P. hirtus* resulted in three mtDNA scaffolds with a total size of 298.6 kb, in which all mtDNA genes were detected except for *nad6*. With three scaffolds we could not determine if the mitochondrial genome is circular. The mtDNA assembly of the obligate biotrophic species *S. taraxaci* extremely fragmented with 14 mtDNA scaffolds with a total size of 39.2 kb coding for all mtDNA genes. Annotation of the mtDNA scaffold of *B. dendrobatidis* JEL423 (DS022322; single scaffold, 175.3 Kb) resulted in the detection of all mtDNA genes except for *nad2* (Additional file 2: Table S1).

### Verification of the *S. endobioticum* linear mtDNA

PCR amplification of the poly-C tailed terminal end to a primer site in the TIR (mtDNA_00787-rv), performed to verify the linearity of the mitogenome, resulted in an amplicon length of expected size (~ 850 bp). Sanger sequencing of the amplicon and alignment to the mitochondrial genome showed a perfect fit to the TIR region.

**Fig. 2:**
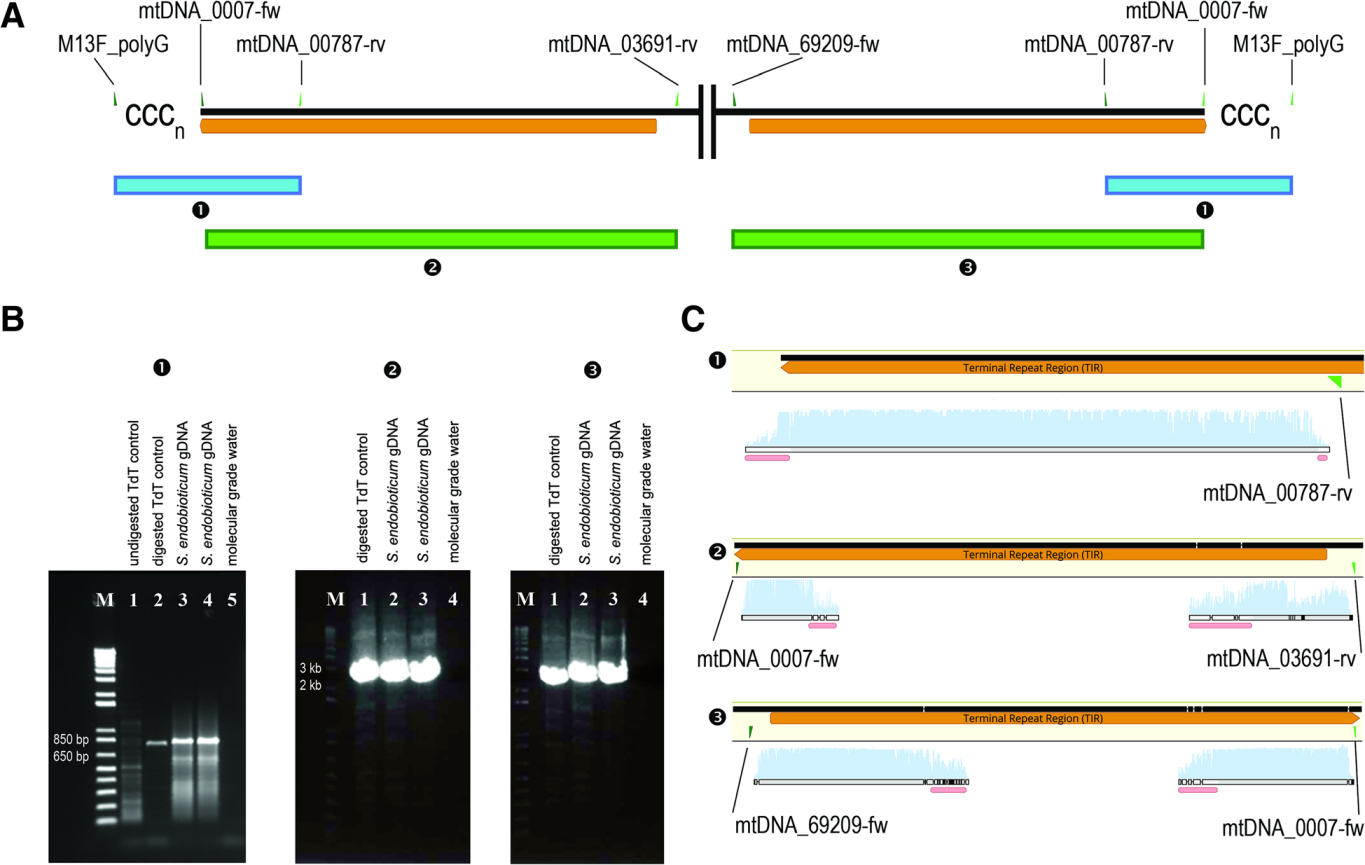
Verification of linearity of the *S. endobioticum* mtDNA. **a** Design of the verification experiments using a graphical representation of the 5′ and 3’ TIRs (orange) and forward and reverse primer sites (green and light green). Three PCR reactions, performed after TdT tailing, are displayed: ❶ M13F_polyG/mtDNA_00787-fw to verify linear conformation of the mtDNA. This reaction takes place at both telomeric ends since the TIRs are inverse orientated. ❷ mtDNA_0007-fw/mtDNA_03691-rv to verify presence of the TIR with its specific flanking sequence on the 5′ end of the mtDNA. ❸ mtDNA_0007-fw/mtDNA_69209-fw to verify presence of the TIR with its specific flanking sequence on the 3′ end of the mtDNA. **b** Gel images corresponding with reactions described under A. The 1 kb-plus size marker (M) was used for amplicon size estimation. **c** Sanger sequence data mapped to the *S. endobioticum* mtDNA corresponding with the reactions described under A. The mtDNA is used as reference, and bases similar to the reference are shown in grey, and differences to the reference are highlighted (black). Phred scores for the individual peaks are shown as blue bars, and low quality data (Phred < 30) is annotated in pink. For reaction 1, the first 800 bases of the 5′ end TIR are shown, whereas for reactions 2 and 3 the full TIR including the specific flanking sequences are shown (~ 4 kb)

Long-range PCR amplification from the telomeric ends, over the TIR sequence, to a 5′ or 3′ specific primer site (mtDNA_03691-rv and mtDNA_69209-fw), performed to verify the presence of identical TIRs and 5′ and 3′ specific sequences, resulted in amplicons of the expected length (~ 3680 bp, and ~ 3710 bp respectively), and Sanger sequence data of the amplicons verified the presence of the repeat on both telomeric ends of the linear molecule (Fig. 2). A linear concatemeric structure of the *S. endobioticum* mitochondrion can be ruled out as no sequence data could be mapped beyond the 5′ and 3′ ends, and no amplicons were obtained using a primer pointing outwards in the TIR (mtDNA_00787-rv). This primer anneals to the TIR and would act as both forward and reverse primer in a PCR reaction bridging the ends of both TIRs should a concatemeric structure exist.

### Bayesian inference of phylogeny

**Fig. 3:**
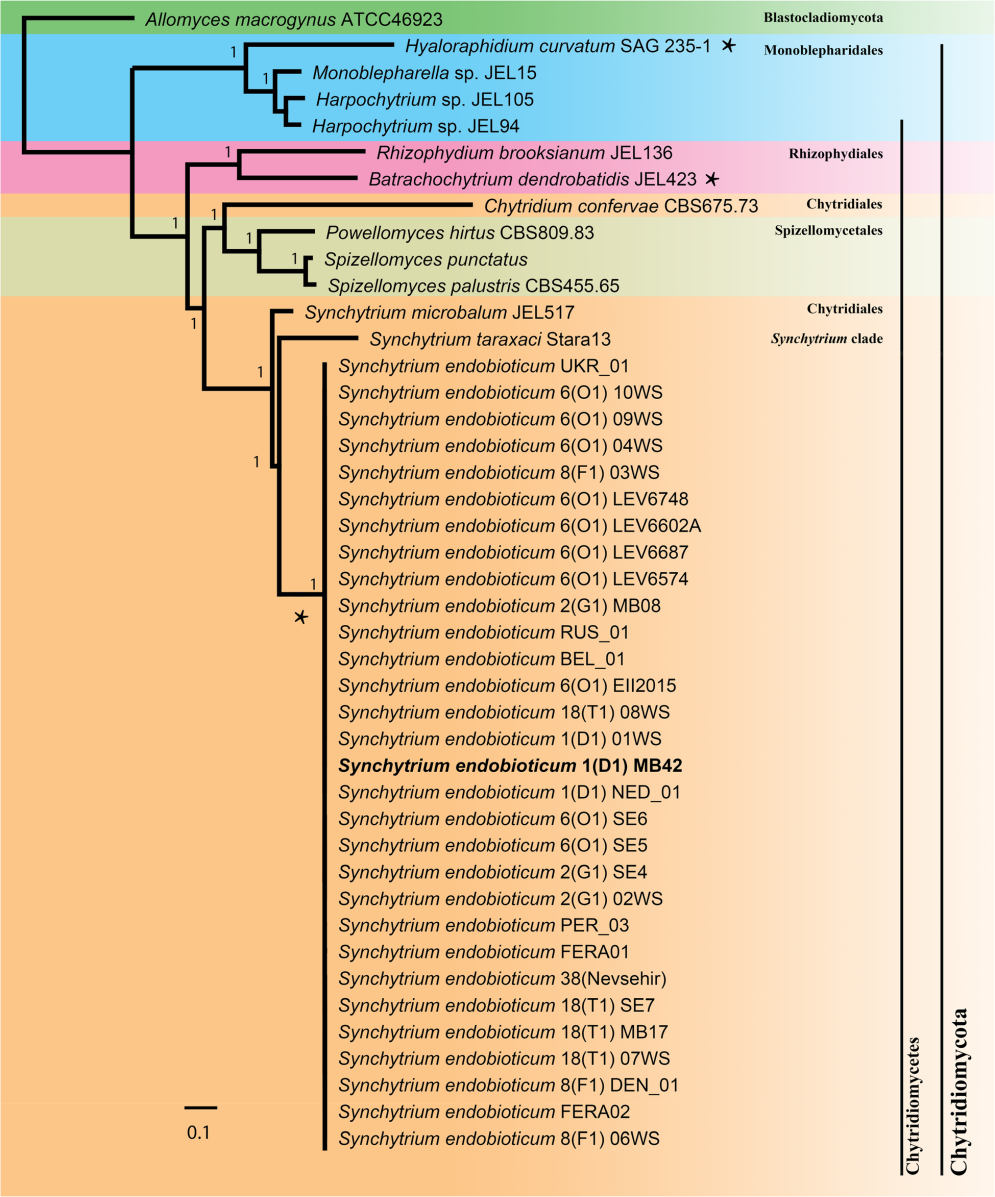
Bayesian tree (GTR model, G + I distributed sites) based on a concatenated alignment of *atp6*, *atp8*, *atp9*, *cob*, *cox1*, *cox2*, *cox3*, *nad1*, *nad2*, *nad3*, *nad4*, *nad4L*, *nad5*, and *nad6* of 43 chytrid isolates with *A. macrogynus* (Blastocladiomycota) serving as outgroup. Bayesian posterior probabilities are displayed at branch nodes. Highlighted in bold is the *S. endobioticum* mtDNA reference isolate MB42. Species with linear mtDNA genomes are indicted with an asterisk “*”

A 16,329 nt alignment (including gaps) was constructed from concatenating individual mitochondrial genes from 44 isolates. The Bayesian phylogeny showed all nodes with a posterior probability of 1.00 (Fig. 3).

### Mitochondrial haplotypes and intraspecies variation

**Fig. 4:**
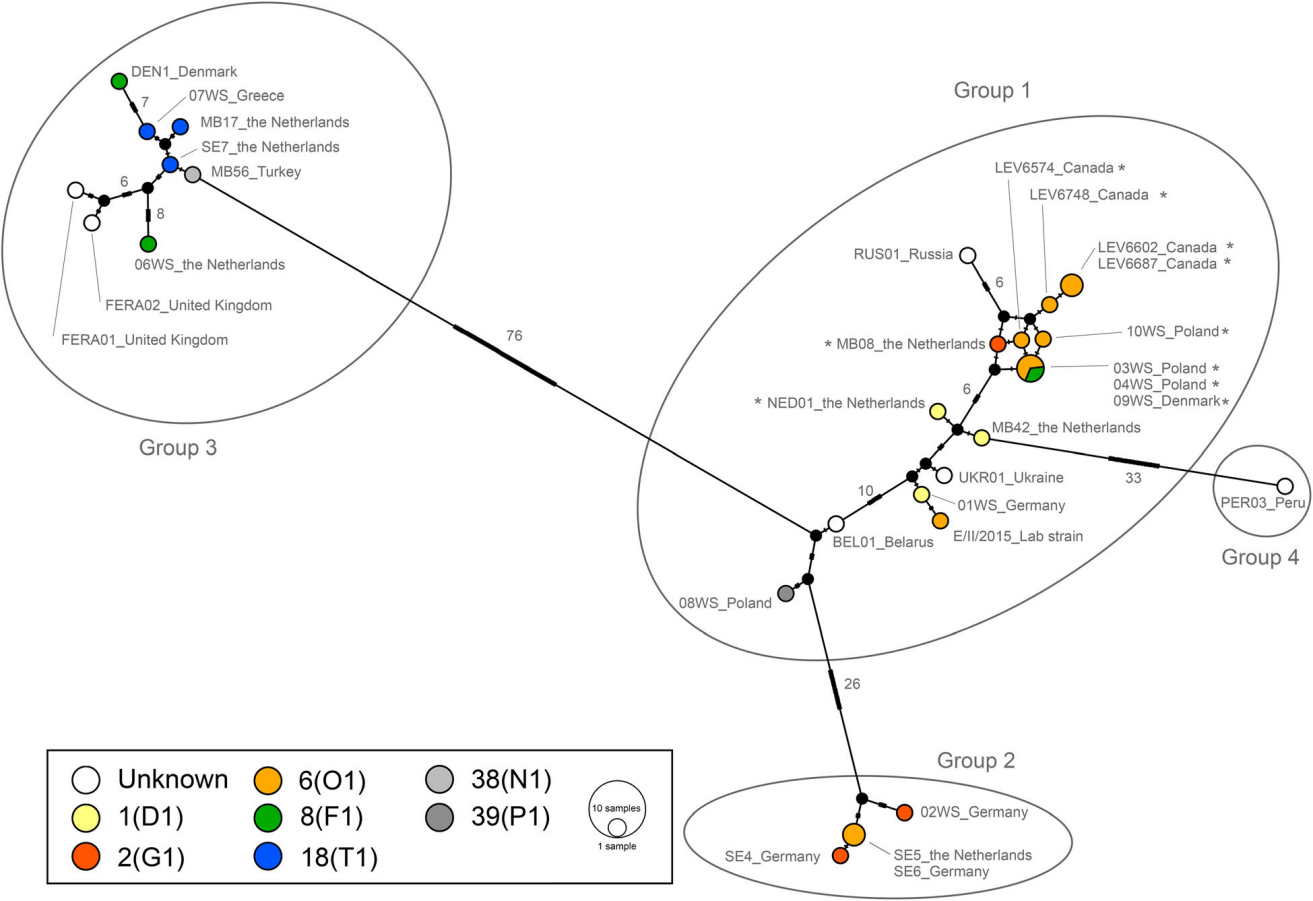
Median Joining haplotype network based on 141 polymorphic sites on the mitochondrial genome of *S. endobioticum*, of which 122 were parsimony-informative. Nodes in the network are coloured based on pathotype identity. Colours are used for pathotypes of major importance in Europe and Canada, whereas a greyscale is used for pathotypes of lesser importance [9]. Black nodes represent hypothetical ancestors. Marks on the branches indicate the number of mutations, and the numbers are shown on branches with > 5 mutations. No signatures for multiple mitochondrial haplotypes were detected for isolates with an asterisk “*”

Reference assembly of 29 additional *S. endobioticum* isolates to the MB42 mtDNA resulted in 72,941 bp to 72,775 bp mitogenomes with 50 to 163,200× mean coverage. Alignment of all 30 *S. endobioticum* mitogenomes resulted in a 73,030 bp alignment (including gaps). Sequence homology between isolates was high at ≥99.21% and sequence variation was found mostly in the intergenic regions (96.8% of the variation). Two polish isolates, 03WS and 04WS, had identical mitochondrial genome sequences. Extraction of variable sites resulted in a 347 bp alignment (including gaps) that contained 122 parsimony-informative sites, which were used to determine mitochondrial haplotypes. Four main groups (i.e. mitochondrial lineages) are distinguished by > 20 mutations (Fig. 4). Clustering based on polymorphic sites in coding sequences of mtDNA genes showed a similar clustering with lower resolution (Additional file 1: Figure S5).

### Composition of *S. endobioticum* populations

Read mapping to the mitochondrial genome showed unexpected variations of SNP frequency for parsimony-informative sites for the majority of the sequenced *S. endobioticum* isolates. Next to the dominant type, low levels of the polymorphic base were observed. For instance, the polymorphism at position 5803 (reference: A, alternative: C) is fixed (> 98%) in nine isolates from group 1, but this SNP is also present in variable frequencies (12–60%) in seven isolates from groups 1, 2 and 3. Similar differences were found for all 122 parsimony-informative sites (Additional file 2: Table S2). In ten isolates from group 1 originating from Canada, Poland and the Netherlands, no signatures for multiple haplotypes were found (Fig. 4). An exception is found in a 40 bp hypervariable intergenic (*atp9*-*cox1*) region containing 13 polymorphic sites which was found to be present in all *S. endobioticum* isolates.

### Pathotype formation

The dynamic process of pathotype formation is clearly illustrated by pathotype 6(O1) isolate E/II/2015. This isolate was obtained after two multiplications of pathotype 1(D1) isolate 01WS on the partial resistant potato cultivar Erika in 2015. Before multiplication on this partial resistant cultivar a clear pathotype 1(D1) phenotype was obtained using the EPPO differential set, whereas after multiplication on the partial resistant potato cultivar, a clear pathotype 6(O1) phenotype was obtained (Table 1). The resulting isolate E/II/2015 is virulent on cultivars Producent and Combi. Repeating the experiment, i.e. multiplying isolate 01WS on cultivar Erika, resulted in the same increased virulence and the same pathotype 6(O1) pathotype profile was obtained. After multiplication, the within isolate variation of E/II/2015 is higher compared to the 01WS isolate.

**Table 1:** Virulence profiles of *S. endobioticum* isolates 1(D1)_01WS and E/II/2015 using differential cultivars described in EPPO PM7/28 (1)

Differential cultivar	1(D1)_01WS	E/II/2015
Deodara	S	S
Tomensa	S	S
Eesterling	S	S
Producent	–	S
Combi	–	S
Saphir	–	–
Delcora	–	±
Miriam	–	–
Karolin	–	–
Ulme	–	–

Susceptible interactions (wart formation) are indicated with “S”, intermediated reactions (sprouts partially proliferated) are indicated with “±”, and resistant interactions are indicated with “-”

## Discussion

We assembled and annotated the mitogenomes of 30 *S. endobioticum* isolates and five additional chytrid species, and compared these to publically available Chytridiomycota mitogenomes. Using the mitogenome sequences, we determined taxonomical relationships of *S. endobioticum* to 14 other chytrid species, and to performed network analyses to identify potential linkages between *S. endobioticum* genotypes, geographical origins and pathotypes.

### The linear mitogenome of *S. endobioticum*

Mitochondrial genomes are typically assumed to have a circular monomeric conformation. However, after the description of a linear mitochondrial genome in *Tetrahymena pyriformis* [22], other linear mitochondrial genomes were reported in fungi, oomycetes, ciliates, Chlorophycean green algae and cnidarians, [23–25]. From our analyses, the *S. endobioticum* mitogenome was found to be linear, encoding a *dpoB* homolog and having two identical TIRs.

In contrast, the mitochondrial genome of the sister species *S. microbalum* is circular, lacks a *dpoB* homolog and comprises only a third of the size of the *S. endobioticum* mitogenome, suggesting recent linearization and expansion in the latter species. Integration of linear mitochondrial plasmids have been hypothesised to be cause of linearization of mitogenomes in medusozoan cnidarians [24] and yeasts [25], and the *dpoB* gene identified in the *S. endobioticum* genome is believed to be reminiscent of such event. Among chytrid fungi, a linear mitochondrial genome is described for *H. curvatum* [26] and *B. dendrobatidis* [27], of which the latter consists of three linear segments, each having inverted repeats at the termini and contains a *dpoB* ortholog.

As was found in other chytrid species [28, 29], *S. endobioticum* contains a reduced set of tRNAs. The tRNA content of the mitochondrial genome determines the genetic code that applies for a given mitogenome. In particular, the presence of trnL(CTA), translating TAG to Leucine instead of introducing a stop codon, indicates that the Chlorophycean Mitochondrial Code (tbl16) applies to the mitogenomes of the species assembled and annotated in this study. The organisation and orientation of the mitochondrial genes, tRNAs and rRNAs are conserved between the linear mtDNA of *S. endobioticum* and the circular mitochondrial genome of *S. microbalum* (Additional file 1: Figure S4). Otherwise, conservation was observed neither in the organisation nor orientation of mitochondrial genes in the Chytridiomycota, underlining the long evolutionary distance among these species.

### Bayesian inference of phylogeny

Clustering of species in their respective orders is identical compared to the nuclear rDNA based phylogeny (18S + 5.8S + 28S subunits) as described by James et al. [30], supporting the polyphyly in the order Chytridiales and monophyly in the genus *Synchytrium* as described by Smith et al. [31]. This further underlines the use and successful application of whole mitochondrial genomes for phylogeny providing the required resolution and robustness in such a wide and scarcely populated evolutionary branch.

### Mitochondrial haplotypes and intraspecies variation

Mitochondrial genotypes did not show direct association with pathotypes or origin (Additional file 2: Table S3). Similar observations were reported by Gagnon et al. [11] using nuclear based microsatellite markers. Isolates of pathotypes 2(G1) and 6(O1) are found both in groups 1 and 2. Also, isolates originating from the Netherlands (e.g. 1(D1)_MB42, 2(G1)_MB08, 6(O1)_SE5, and 18(T1)_MB17), are represented in groups 1, 2 and 3. European pathotype 6(O1) isolates are found in both groups 1 and 2, and pathotype 8(F1) isolates are present in clusters 1 and 3. Nevertheless, pathotype 18(T1) isolates are found exclusively in group 3 even though they originate from different countries. Similarly, pathotype 6(O1) isolates originating from Prince Edward Island, Canada are found only in group 1.

The capacity of *S. endobioticum* for natural dispersion is limited, and the pest is mainly spread as a result of human crop exchange and agricultural activities [2, 32]. Our observation that *S. endobioticum* isolates with identical multi-gene mitochondrial haplotypes can have a global distribution corroborates with distribution as a result of human activities. If regional or worldwide distribution would pre-date agricultural development, a much larger variation in mitochondrial haplotypes between isolates from distant locations would be expected. Based on the mitochondrial lineages identified, we conclude that *S. endobioticum* has been introduced at least three times in Europe, and that from those introductions the disease has spread in Europe and to other continents. Isolates from pathotype 18(T1), identified first in Germany in 1978 [33], cluster in mtDNA group 3, suggesting that the emergence of this pathotype was the result of a new introduction opposed to the gain of increased virulence of isolates already present in Europe. The forth group is represented by a single isolate originating from Peru and fits well with the hypothesis that more genetic diversity can be expected from the Andean region, the presumed centre of origin of this pathogen.

Pathotype formation in *S. endobioticum* is a dynamic and ongoing process. This is supported by the occurrence of the same pathotypes in different mitochondrial lineages, e.g. pathotypes 2(G1) and 6(O1) being found in both mitochondrial groups 1 and 2. Our data suggests that the emergence of those pathotypes have occurred independently in a different genetic background. Different SNP frequencies in read mappings demonstrate the presence of more than one mitochondrial haplotype in the majority of isolates. This was not observed in the other chytrid species sequenced in this study, with the exception of *S. taraxaci*; a closely related species with a similar obligate biotrophic pathogenic life style on its host dandelion. The number of SNPs was not high enough for the size of the fragments to assemble the individual haplotypes for isolates MB42 and LEV6574 using long read PacBio sequences and the recently published Canu assembler [34]. The composition of the *S. endobioticum* communities of different genotypes seem to be conserved. Isolates from group 1 typically portray low levels of diversity, whereas isolates from group 3 have high levels of diversity (Additional file 1: Figure S6). Ten isolates clustering together in group 1 are strikingly scarce in diversity, apart from the 40 bp *atp9*-*cox1* intergenic region which found to be variable in all isolates. We suspect that this region could represent the origin of replication which may involve an RNA intermediate as was described for linear chromosomal fragments [35].

Inoculation experiments show that the use of semi-resistant potato cultivars triggers a rapid shift (increase) in virulence, which was associated with a shift in the mitochondrial haplotypes found in the isolate. In fact an increase in diversity was observed. Most likely this variation was already present, but below the detection limit of the sequencing depth. We postulate that this increased virulence was the result of a shift in the heterogeneous 1(D1) population of *S. endobioticum* due to the selection of virulent genotypes already present in the isolate. Functional variation could be present on the nuclear genome as we found evidence for genetic heterogeneity not only on the mitochondrial genome but also on some nuclear scaffolds in all sequenced isolates (data not shown). The alternative hypothesis that many new mutations could be induced during the two multiplications seems highly unlikely. The possible rapid increase in virulence due to selection of the population underlines that caution is needed when adopting the deployment of *R* genes in a control strategy, in particular when they provide partial or QTL based resistance.

## Conclusions

The mitochondrial genome of *S. endobioticum* and other Chytridiomycota provides insight into the evolution of mitochondria. Of special interest are the linearization of what is often assumed to be a circular genome which happened multiple times during the evolution of Chytridiomycota, the extremely variable size range (19,5 to 298,5 Kb), and the remarkable high GC content for the intergenic regions (close to 70%). The mitochondrial genome also provides insight in the more recent history of this plant pathogen. From this study we conclude there were at least three independent introductions in Europe, most likely as the result of human activities. With one of those introductions, pathotype 18(T1) was introduced into Europe and so far this pathotype seems to be confined to Europe. A fourth mtDNA lineage obtained from a Peruvian isolate represents a genotype not found in other *S. endobioticum* isolates sequenced so far. Mitochondrial data shows that *S. endobioticum* isolates should not be considered as a single genotype, but rather populations of different genotypes. The observed population diversity seems to be consistent in the four different mitochondrial lineages identified in this study. Also, we conclude that pathotypes 2(G1) and 6(O1) have emerged at least twice independently, most likely by selection of virulent genotypes from diverse population as was observed for a pathotype 6(O1) derivative from a pathotype 1(D1) isolate.

Our data indicate that characterization by pathotyping alone is insufficient for characterising isolates, as underlying diversity may remain unnoticed. To keep track of genetic drift and selection of individuals from such populations we recommend sequencing of isolates before and after each multiplication, particularly when (partial) resistant potato cultivars are used. Obtaining genetically pure cultures may not be possible since *S. endobioticum* is an obligate biotroph. This situation may be similar for other plant pathogens in this genus as illustrated by the fact that a heterogeneous genotypic profile in the mtDNA of *S. taraxaci* was observed. To our knowledge *S. endobioticum* represents a unique case where although the number of (new) outbreaks of the disease is low, the genetic diversity is high.

## Methods

### Fungal material

A total of 30 *S. endobioticum* isolates were included in this study covering eleven different pathotypes and six isolates with unknown pathotype identity. Material was collected from countries in Europe, North America, South America and Asia (Table 2). Pathotype identification for the Canadian isolates was performed according to the Glynne-Lemmerzahl method [7]. The samples were provided either as wart material, resting spores or DNA extracts. When provided with wart material, resting spores were extracted following the procedure described by Bonants et al. [36]. For the Canadian isolates, resting spores were collected in a 37 μm sieve and treated with a 1/10 dilution of viscozyme (Sigma-Aldrich) in 100 mM phosphate buffered saline at pH 5.0 at 30 °C for 16 h. Spores were layered onto a 50% solution of sucrose and centrifuging at 2000 x *g* for 20 min. Spores were collected in a sieve, washed, and further purified by pelleting through a 90% solution of Percoll (Sigma-Aldrich) at 1000 x g for 5 min. Cultures of *Chytridium confervae* (CBS 675.73), *Powellomyces hirtus* (CBS 809.83), *Spizellomyces palustris* (=*Phlyctochytrium palustre*) (CBS 455.65) and *Synchytrium microbalum* (JEL517) were maintained on ARCH medium and ARCH broth (Broth: peptone 2 g (Oxoid), malt extract 3 g (Oxoid), glucose 5 g ·L^− 1^, pH 6. The medium contains 8 g·L^− 1^ agar (Oxoid)) at 21 °C. Dandelion leaves containing *Synchytrium taraxaci* spores were collected from a meadow in Bennekom the Netherlands (GPS coordinates: 51.997314, 5.662961) in April 2015. *S. taraxaci* spores were extracted by gentle disruption of epidermal leaf tissue using a pipette tip. Extracted spores were washed with tap water and stored at − 20 °C until DNA extraction.

**Table 2:** Fungal material used in this study

*S. endobioticum* isolates				
Pathotype, based on		isolate	origin	mtDNA accession
EPPO PM7/28(1)	Additional cultivars^a^			
1(D1)	n.a.	MB42	Langenboom, the Netherlands	ERZ668671
1(D1)	n.a.	1/2007/D1 (01WS)	Germany	ERZ668651
1(D1)	n.a.	NED01	Brabant, the Netherlands	ERZ668673
2(G1)	n.a.	4/2005/G1 (02WS)	Germany	ERZ668652
2(G1)	n.a.	MB08	Mussel, the Netherlands	ERZ668669
2(G1)	n.a.	BBA 2(G1) 09–04 (SE4)	Germany	ERZ668676
6(O1)	3(M1)	PL28/2007/2 (04WS)	Poland	ERZ668654
6(O1)	P40	DK17/2015 (09WS)	Denmark	ERZ668658
6(O1)	P41	PL2/2015 (10WS)	Poland	ERZ668659
6(O1)	n.a.	E/II/2015	Laboratory isolate obtained after two multiplications of isolate 1/2007/D1 (01WS) on cultivar Erika	ERZ668662
6(O1)	n.a.	LEV6574	St. Eleanor’s, Prince Edward Island, Canada	ERZ668665
6(O1)	n.a.	LEV6602	Augustine Cove, Prince Edward Island, Canada	ERZ668666
6(O1)	n.a.	LEV6687	New Annan, Prince Edward Island, Canada	ERZ668667
6(O1)	n.a.	LEV6748	New Glasgow, Prince Edward Island, Canada	ERZ668668
6(O1)	n.a.	HLB 6(O1) 02–06 (SE5)	the Netherlands	ERZ668677
6(O1)	n.a.	BBA 6(01) 05_8.3 (SE6)	Germany	ERZ668678
8(F1)	n.a.	DEN01	Jylland, Denmark	ERZ668661
8(F1)	n.a.	3/2005/F1 (06WS)	The Netherlands	ERZ668655
8(F1)	2(Ch1)	2/2005/Ch1 (03WS)	Poland	ERZ668653
18(T1)	n.a.	GR2/2015 (07WS)	Greece	ERZ668656
18(T1)	n.a.	MB17	Borgercompagnie, the Netherlands	ERZ668670
18(T1)	n.a.	HLB P18(T1) -02-06 (SE7)	Borgercompagnie, the Netherlands	ERZ668679
38(Nevsehir)	n.a.	MB56	Nevsehir, Turkey	ERZ668672
39(P1)	n.a.	PL69/2009 (08WS)	Piekielnik, Poland	ERZ668657
unknown	n.a.	BEL01	Belarus	ERZ668660
unknown	n.a.	FERA01	United Kingdom	ERZ668663
unknown	n.a.	FERA02	United Kingdom	ERZ668664
unknown	n.a.	PER03	Aco Paucartambo, Pasco, Peru	ERZ668674
unknown	n.a.	RUS01	St. Petersburg, Russian Federation	ERZ668675
unknown	n.a.	UKR01	Ukraine	ERZ668680
Other chytrid strains				
species	strain	Origin	ITS accession	mtDNA accession(s)
*Chytridium confervae*	CBS 675.73	Canada	MH660417	ERZ681044
*Powellomyces hirtus*	CBS 809.83	the Netherlands	MH660418	ERZ681050
*Spizellomyces palustris*	CBS 455.65	Germany	MH660420	ERZ681046
*Synchytrium microbalum*	JEL517	Hancock Co., Maine, USA	MH660419	ERZ681045
*Synchytrium taraxaci*	Stara13	Bennekom, the Netherlands	MH660421	ERZ681051

a. In this paper, pathotype identities are used based on the potato cultivar differential set as presented in EPPO standard PM7/28 (1). In some cases, additional cultivars were used resulting in a further differentiation of pathotype identity (n.a. when not applicable)

### Multiplication of pathotype 1(D1) isolate 01WS on potato cultivar Erika

Eye pieces of cultivar Erika were inoculated with winter sporangia of pathotype 1(D1) isolate 01WS. After two months of incubation, maturated winter sporangia from small warts were removed and used for a second round of inoculation on cultivar Erika. After each multiplication the virulence was checked on a differential set of potato cultivars. When wart development on cultivars known to be resistant to pathotype 1(D1) was observed, a full virulence profile was determined on an extended set of potato cultivars. After two multiplications, isolate 01WS produced a pathotype 6(O1) virulence spectrum and the isolate was renamed to E/II/2015. The experiment was repeated at a different moment with similar results.

### DNA extraction

A total of 200 μl suspensions containing ≥5000 *S. endobioticum* resting spores were homogenised in a Hybaid Ribolyser multiple bead beater (Thermo Electron Corporation, the Netherlands) at 5000 bpm for 100 s using three stainless steel beads (3.2 mm). For the WS and E/II/2015 isolates, resting spores were homogenised using a combination of vortexing and ultrasound sonication. Subsequently the Ultra Clean Soil DNA kit (MoBio) was used according to manufacturer’s instructions to extract genomic DNA. For the Canadian isolates, DNA was extracted from resting spores as previously described [14]. Genomic DNA of the culturable chytrids was extracted from fungal cells grown in 8 mL ARCH broth, and 50 μL spore suspensions containing *S. taraxaci* spores using the Wizard Magnetic DNA Purification System for Food (Promega) following manufacturer’s instructions. When sufficient starting material was available multiple DNA extracts were generated per isolate. The nuclear ITS region was amplified and sequenced to verify the species identity using primers ITS4 [37] and Chy18S-269A [31] as a check before Next Generation Sequencing (NGS).

### Quantification of obligate biotrophs

*S. endobioticum* DNA was quantified using a *S. endobioticum* specific real-time PCR [38]. For the quantification of *S. taraxaci*, a species specific real-time PCR was designed based on publicly available ITS1–5.8S-ITS2 sequences covering 20 *Synchytrium* species (Additional file 1: Figure S7). DNA extracts with exponential amplification curves and Cq values below 30 were selected for NGS.

### Next generation sequencing

NGS data was generated using one or more of three sequencing platforms: HiSeq 2500 (Illumina), MiSeq (Illumina), PacBio RSII (Pacific Biosciences) (Additional file 2: Table S4).

### Assembly and annotation of the *S. endobioticum* mitochondrial genome

A consensus mitochondrial sequence of *S. endobioticum* isolate MB42 pathotype 1(D1) was created using the results of three independent assemblies with two different datasets. In the first assembly, Illumina HiSeq reads were mapped to the *S. endobioticum* MB42 reference genome (van de Vossenberg et al., unpublished data) in CLC genomics workbench v8.0.2. Scaffolds coverage > 50 times above the expected coverage for single copy nuclear regions were selected and annotated using Mfannot with the Chlorophycean Mitochondrial Code (tbl 16) [39]. Contigs containing an Mfannot annotation and/or those with an e-value of <1e-20 when compared by blastn to other publically available chytrid mitochondrial genome sequences, were selected. In the second assembly, the GRAbB assembly tool [40] was used to reconstruct the *S. endobioticum* mitochondrial genome using the putative *S. endobioticum* scaffolds identified in the first assembly, as bait for the raw genomic library. The reads collected by the final iteration of the GRAbB run were used to perform de novo assemblies using three different assemblers using SPAdes v3.6 [41], Velvet v1.2.10 [42] and Edena v3.131028 [43]. Overlaps between contigs produced by the assemblers were manually identified and merged using the helper tools distributed with the GRAbB assembler until all regions were included in a single contig. Raw reads were mapped back to the final sequence and errors were corrected with Pilon v1.19 [44] iteratively until no further errors were identified. In the third assembly, the mitochondrial genome of *S. endobioticum* MB42 was assembled with Celera Assembler version 8.2 [45] using the PacBio corrected reads [35]. Using the largest scaffold as a backbone sequence, all putative mitochondrial scaffolds from the three independent assemblies were assembled in Geneious R10 [46] using default parameters. Conflicts were resolved using a majority rule. In addition, the Illumina HiSeq and PacBio reads from *S. endobioticum* MB42 were mapped to this consensus sequence to further identify and resolve conflicts using the quality based majority rule resulting in a final consensus *S. endobioticum* mitochondrial genome. This final consensus was verified using the PacBio reads mapping to the consensus, which were assembled using the Canu assembler [34]. The final consensus sequence was then annotated using Mfannot. Putative mitochondrial genes, tRNAs and rRNAs were manually curated. Amino acid sequences from predicted gene models were compared to those in InterProScan [47] and the NCBI nr database by blastp and those with an e-value of <1e-20 with a known predicted function were kept as valid and those that did not meet these criteria were filtered out.

### Molecular verification of mitochondrial genome linearity

A TdT tailing method based on [48, 49] was used as a method to verify that the linear conformation of the *S. endobioticum* mitochondrial genome. Positive and negative controls (i.e. synthetic oligo resembling the mitochondrial genome sequence in a linear and circular form respectively, see (Additional file 1: Figure S8) were included to monitor the effectiveness of the TdT tailing. Sample DNA (30–45 ng/μL) was denatured at 95 °C for 5 min, placed on ice and then immediately used in a 25 μl of 3′-TdT reaction consisting of 1× TdT buffer (TaKaRa), 0.01% BSA, 300 μmol of dCTP (TaKaRa), 7 U of TdT enzyme (TaKaRa), and 1 μL of sample DNA. TdT tailing was performed at 37 °C for 30 min, followed by the inactivation of the TdT enzyme at 65 °C for 10 min. Linearity of the mtDNA was tested by PCR amplification using the primers listed in Table 3. Amplification from the poly-C 3′ ends of the mtDNA into the terminal inverted repeat (TIR) was performed with primers M13F_polyG and mtDNA_00787-rv using a reaction mix containing 1× GoTaq flexi buffer (Promega), 1.5 mM MgCl_2_, 200 μM dNTP mix, 300 nM of each primer, 1 U Taq polymerase (Promega), and 1 μL poly-C tailed genomic DNA. Cycling conditions were as follows: 2 min at 95 °C, 35× (30 s at 95 °C, 30 s 60 °C, 1 min 72), 5 min 72 °C. The presence of TIRs on both the 5′ and 3′ end of the linear mtDNA was verified by Long-Range PCR from the telomeric ends of the mtDNA over the TIR to both site specific ends with primer pairs mtDNA_0007-fw/mtDNA_03691-rv for the 5′ end, and mtDNA_0007-fw /mtDNA_69209-fw for the 3′ end. Reaction mixes for the latter two primer combinations consisted of 1× GoTaq Long Master Mix (Promega), 300 nM of each primer, and 1 μL poly-C tailed genomic DNA. Cycling conditions were as follows: 2 min at 95 °C, 35× (30 s at 95 °C, 30 s 60 °C, 3.5 min 72), 5 min 72 °C. After electrophoresis, PCR products were excised from the gel, purified with the Wizard SV gel and PCR clean-up system (Promega) and submitted for sequencing using the generic M13F sequencing primer, and specific mtDNA amplification primers in separate reactions. The resulting sequence data were mapped to the assembled and annotated *S. endobioticum* consensus mitochondrial genome in Geneious.

**Table 3:** Amplification and sequencing primers used for verification of linearity mtDNA genome

Primer name	Primer sequence (5′ - 3′ direction)
M13F_polyG	TGTAAAACGACGGCCAGTGGGGGGGGGGGGGGGG
M13F	TGTAAAACGACGGCCAGT
mtDNA_0007-fw	GTTTTCTTAGGCCCATCCT
mtDNA_00787-rv	CAGTTTTCTCTAGGGTTCCA
mtDNA_03691-rv	AATAGACTATCAGAACCACCAAG
mtDNA_69209-fw	CTCAAACACAGAAATTAACAACG

### Assembly and annotation mitochondrial genome of other species

Illumina NGS data of other chytrid species: *C. confervae*, *P. hirtus*, *S. palustris*, *S. microbalum* and *S. taraxaci* were used for de novo assembly using CLC genomics workbench (word size: automatic, bubble size: automatic, scaffolding: on) and scaffolds were updated using a read mapping approach (length fraction: 0.5, similarity fraction: 0.8) (van de Vossenberg et al., unpublished data). Scaffolds with relative high coverage were selected and annotated using Mfannot (tbl 16) as described above for *S. endobioticum* MB42.

### Bayesian inference of phylogeny

NGS datasets from other isolates of *S. endobioticum* were mapped to the annotated *S. endobioticum* MB42 mitochondrial genome in CLC genomics workbench (length fraction: 0.8, similarity fraction: 0.9, non-specific match: map random, resolve conflicts: quality based) and regions with ≥5× coverage were extracted. Annotations from the *S. endobioticum* MB42 reference mitochondrial genome were transferred to the consensus sequence. Coding sequences of 14 mitochondrial genes were extracted from the consensus sequences and these were aligned with MAFFT v7.308 [50] to coding sequences of the same genes from publically available complete chytrid mitochondrial genomes (Additional file 2: Table S1). *Allomyces macrogynus* ATCC46923 (NC_001715), *Harpochytrium* sp. JEL94 (AY182005), *Harpochytrium* sp. JEL105 (AY182006), *Hyaloraphidium curvatum* SAG 235–1 (AF402142), *Monoblepharella* sp. JEL15 (NC_004624), *Rhizophydium brooksianumb* JEL136 (NC_003053), and *Spizellomyces punctatus* (NC_003052). In addition, a mitochondrial scaffold was identified from the *Batrachochytrium dendrobatidis* JEL423 genome (GCA_000149865.1) by blastn (e-value <1e-20) to other publically available chytrid mitochondrial genome sequences. *B. dendrobatidis* mitochondrial scaffold DS022322 was annotated using Mfannot (tbl 16). Alignments were concatenated and phylogenies were constructed using Bayesian inference (BI) [51]. Models for nucleotide substitution were obtained using JModelTest 2.1.10 [52] which was run on default settings and the best nucleotide substitution model, according to AIC, AICc, BIC and DT, was the GTR model with gamma distribution and estimation of invariable sites (GTR + G + I). BI was performed with the MrBayes v3.2.6 plugin incorporated in Geneious with a random starting tree and four Monte Carlo Markov Chains for 10^6^ generations. Trees were sampled every 200 generations, and the first 10^5^ generations were discarded as burn-in. Remaining trees were combined to generate a 50% majority rule consensus tree with posterior probabilities.

### Mitochondrial haplotypes and intraspecies variation

The mitochondrial genomes of 30 *S. endobioticum* isolates were aligned with MAFFT v7.308 to estimate the relationship between *S. endobioticum* isolates. Low coverage terminal sequences (< 5× coverage) were trimmed from the alignment and variable sites were extracted with “Show variable sites only” from the online fasta sequence toolbox FaBox [53]. A Median Joining (MJ) network (ε = 0, uninformative sites = masked) was calculated with Popart v1.7 [54]. Variation within a isolate for a given polymorphic site was determined for informative sites in the MJ network with the basic variant calling tool, including only paired reads but allowing non-specific matches to include TIRs, in CLC genomics workbench (min_coverage_ = 5×, min_count_ = 2, min_frequency_ = 10%, min_base quality_ = 20, direction frequency filter = 5%).

## Additional files

Additional file 1: **Figure S1.** PacBio read mapping to the *S. endobioticum* mtDNA. **Figure S2.** Dotplot internal repeat structure TIRs **Figure S3.** Dotplot repeat in AT-rich region containing *dpoB* gene **Figure S4.** Interspecies comparison of organisation and orientation of mitochondrial genes, tRNAs and rRNAs **Figure S5.** Haplotype network based on CDS of mtDNA genes *S. endobioticum* **Figure S6.** Within-strain diversity in haplotype network based on polymorphic sites of the *S. endobioticum* mtDNA **Figure S7.** *Synchytrium taraxaci* Taqman assay **Figure S8.** TdT-tailing controls. (DOCX 2313 kb)

Additional file 2: **Table S1.** mitogenomes and mitochondrial genes included in the Bayesian inference (BI) of phylogeny **Table S2.** SNP percentages of polymorphic sites relative to the *S. endobioticum* 1(D1)_MB42 mitogenome **Table S3.** Mitochondrial haplotype classification of *S. endobioticum* isolates based on informative sites in coding sequences of seven mitochondrial genes **Table S4.** Fungal materials and NexGen sequence information. (XLSX 56 kb)

## Abbreviations

AIC: Akaike information criterion
AICc: Akaike information criterion corrected for small sample sizes
ATP: Adenosine triphosphate
BI: Bayesian inference
BIC: Bayesian information criterion
DT: Decision-theoretic performance-based
GTR: Generalised time-reversible nucleotide substitution model
HEG: Homing endonuclease gene
MJ: Median joining
mtDNA: Mitochondrial DNA
SNP: Single nucleotide polymorphism
TdT: Terminal deoxynucleotidyl transferase
TIR: Terminal inverted repeat

## References

[1] Smith IM, McNamara DG, Scott PR, Holderness M, Burger B (1997). Quarantine pests for Europe - data sheets on quarantine pests for the European Union and for the European and Mediterranean plant protection organization. 2.

[2] Obidiegwu JE, Flath K, Gebhardt C (2014). Managing potato wart: a review of present research status and future perspective. Theor Appl Genet.

[3] Laidlaw WMR (1985). A method for the detection of the resting sporangia of potato wart disease (Synchytrium endobioticum) in the soil of old outbreak sites. Potato Res.

[4] Przetakiewicz J (2015). The viability of winter sporangia of Synchytrium endobioticum (Schilb.) perc. From Poland. Am J Potato Res.

[5] Hampson MC (1993). History, biology and control of potato wart disease in Canada. Can J Plant Pathol.

[6] Federal Select Agent Program, Select Agents and Toxins List: United States Department of Agriculture;Centers for Disease Control and Prevention; 2018 [cited 2018 25 April]. Available from: www.selectagents.gov/selectagentsandtoxinslist.html.

[7] Schilberszky K (1896). Ein neuer Schorfparasit der Kartoffelknollen. Ber Deut Botan Ges.

[8] Braun HC (1942). Biologische Spezialisierung bei Synchytrium endobioticum (Schilb.). Zeitschrift für Pflanzenkrankheiten (Pflanzenpathologie) und Pflanzenschutz.

[9] Baayen RP, Cochius G, Hendriks H, Meffert JP, Bakker J, Bekker M (2006). History of potato wart disease in Europe – a proposal for harmonisation in defining pathotypes. Eur J Plant Pathol.

[10] Przetakiewicz J (2015). First report of new Pathotype 39(P1) of Synchytrium endobioticum causing potato wart disease in Poland. Plant Dis.

[11] Gagnon MC, van der Lee TA, Bonants PJ, Smith DS, Li X, Levesque CA (2016). Development of polymorphic microsatellite loci for potato wart from next-generation sequence data. Phytopathology.

[12] Friedman JR, Nunnari J (2014). Mitochondrial form and function. Nature.

[13] Taanman J-W (1999). The mitochondrial genome: structure, transcription, translation and replication. Biochim Biophys Acta.

[14] Smith DR (2016). The past, present and future of mitochondrial genomics: have we sequenced enough mtDNAs?. Brief Funct Genomics.

[15] Gilbert MTP, Drautz DI, Lesk AM, Ho SYW, Qi J, Ratan A (2008). Intraspecific phylogenetic analysis of Siberian woolly mammoths using complete mitochondrial genomes. Proc Natl Acad Sci.

[16] Ma C, Yang P, Jiang F, Chapuis MP, Shali Y, Sword GA (2012). Mitochondrial genomes reveal the global phylogeography and dispersal routes of the migratory locust. Mol Ecol.

[17] Janssen T, Karssen G, Verhaeven M, Coyne D, Bert W (2016). Mitochondrial coding genome analysis of tropical root-knot nematodes (Meloidogyne) supports haplotype based diagnostics and reveals evidence of recent reticulate evolution. Sci Rep.

[18] Simon C, Frati F, Beckenbach A, Crespi B, Liu H, Flook P (1994). Evolution, weighting, and phylogenetic utility of mitochondrial gene sequences and a compilation of conserved polymerase chain reaction primers. Ann Entomol Soc Am.

[19] Hebert PDN, Cywinska A, Ball SL, deWaard JR (2003). Biological identifications through DNA barcodes. Proc R Soc Lond Ser B Biol Sci.

[20] Karling JS (1964). Synchytrium.

[21] Longcore JE, Simmons DR, Letcher PM (2016). Synchytrium microbalum sp. nov. is a saprobic species in a lineage of parasites. Fungal biology.

[22] Suyama Y, Miura K (1968). Size and structural variations of mitochondrial DNA. Proc Natl Acad Sci U S A.

[23] Nosek J, Tomaska L, Fukuhara H, Suyama Y, Kovac L (1998). Linear mitochondrial genomes: 30 years down the line. Trends genet.

[24] Burger G, Gray MW, Lang BF (2003). Mitochondrial genomes: anything goes. Trends Genet.

[25] Lavrov DV, Pett W (2016). Animal mitochondrial DNA as we do not know it: mt-genome organization and evolution in Nonbilaterian lineages. Genome Biol Evol.

[26] Forget L, Ustinova J, Wang Z, Huss VAR, Lang BF (2002). Hyaloraphidium curvatum: a linear mitochondrial genome, tRNA editing, and an evolutionary link to lower fungi. Mol Biol Evol.

[27] O’Hanlon SJ, Rieux A, Farrer RA, Rosa GM, Waldman B, Bataille A (2018). Recent Asian origin of chytrid fungi causing global amphibian declines. Science.

[28] Bullerwell CE, Forget L, Lang BF (2003). Evolution of monoblepharidalean fungi based on complete mitochondrial genome sequences. Nucleic Acids Res.

[29] Laforest M, Bullerwell CE, Forget L, Lang BF (2004). Origin, evolution, and mechanism of 5′ tRNA editing in chytridiomycete fungi. RNA.

[30] James TY, Letcher PM, Longcore JE, Mozley-Standridge SE, Porter D, Powell MJ (2006). A molecular phylogeny of the flagellated fungi (Chytridiomycota) and description of a new phylum (Blastocladiomycota). Mycologia.

[31] Smith DS, Rocheleau H, Chapados JT, Abbott C, Ribero S, Redhead SA (2014). Phylogeny of the genus Synchytrium and the development of TaqMan PCR assay for sensitive detection of Synchytrium endobioticum in soil. Phytopathology.

[32] Hampson MC (1996). A qualitative assessment of wind dispersal of resting spores of Synchytrium endobioticum, the causal agent of wart disease of potato. Plant Dis.

[33] Stachewicz H (1978). Nachweis eines neuen Biotypen des Kartoffelkrebserregers Synchytrium endobioticum (Schilb.) Perc. in der DDR. Nachrichtenblatt für den Pflanzenschutz in der DDR.

[34] Koren S, Walenz BP, Berlin K, Miller JR, Bergman NH, Phillippy AM. Canu: scalable and accurate long-read assembly via adaptive k-mer weighting and repeat separation. Genome Res. 2017; 10.1101/gr.215087.116 PMC5411767 28298431

[35] Berlin K, Koren S, Chin CS, Drake JP, Landolin JM, Phillippy AM (2015). Assembling large genomes with single-molecule sequencing and locality-sensitive hashing. Nat Biotechnol.

[36] Bonants PJM, van Gent-Pelzer MPE, van Leeuwen GCM, van der Lee TAJ (2015). A real-time TaqMan PCR assay to discriminate between pathotype 1 (D1) and non-pathotype 1 (D1) isolates of Synchytrium endobioticum. Eur J Plant Pathol.

[37] White TJ, Bruns T, Lee SJ, Taylor JL, IMAGDHSJJW TJ (1990). Amplification and direct sequencing of fungal ribosomal RNA genes for phylogenetics. PCR protocols: a guide to methods and applications.

[38] van Gent-Pelzer MPE, Krijger M, Bonants PJM (2010). Improved real-time PCR assay for detection of the quarantine potato pathogen, Synchytrium endobioticum, in zonal centrifuge extracts from soil and in plants. Eur J Plant Pathol.

[39] Mfannot: University of Montreal; [cited 2018 25 April]. Available from: http://megasun.bch.umontreal.ca/cgi-bin/mfannot/mfannotInterface.pl.

[40] Brankovics B, Zhang H, van Diepeningen AD, van der Lee TA, Waalwijk C, de Hoog GS (2016). GRAbB: selective assembly of genomic regions, a new niche for genomic research. PLoS Comput Biol.

[41] Bankevich A, Nurk S, Antipov D, Gurevich AA, Dvorkin M, Kulikov AS (2012). SPAdes: a new genome assembly algorithm and its applications to single-cell sequencing. J Comput Biol.

[42] Zerbino DR, Birney E (2008). Velvet: algorithms for de novo short read assembly using de Bruijn graphs. Genome Res.

[43] Hernandez D, Francois P, Farinelli L, Osteras M, Schrenzel J (2008). De novo bacterial genome sequencing: millions of very short reads assembled on a desktop computer. Genome Res.

[44] Walker BJ, Abeel T, Shea T, Priest M, Abouelliel A, Sakthikumar S (2014). Pilon: an integrated tool for comprehensive microbial variant detection and genome assembly improvement. PLoS One.

[45] Koren S, Harhay GP, Smith TPL, Bono JL, Harhay DM, McVey SD (2013). Reducing assembly complexity of microbial genomes with single-molecule sequencing. Genome Biol.

[46] Kearse M, Moir R, Wilson A, Stones-Havas S, Cheung M, Sturrock S (2012). Geneious basic: an integrated and extendable desktop software platform for the organization and analysis of sequence data. Bioinformatics.

[47] Jones P, Binns D, Chang HY, Fraser M, Li W, McAnulla C (2014). InterProScan 5: genome-scale protein function classification. Bioinformatics.

[48] Hikosaka K, Tsuji N, Watanabe Y, Kishine H, Horii T, Igarashi I (2012). Novel type of linear mitochondrial genomes with dual flip-flop inversion system in apicomplexan parasites, Babesia microti and Babesia rodhaini. BMC Genomics.

[49] Ogedengbe ME, Qvarnstrom Y, AJd S, Arrowood MJ, Barta JR (2015). A linear mitochondrial genome of Cyclospora cayetanensis (Eimeriidae, Eucoccidiorida, Coccidiasina, Apicomplexa) suggests the ancestral start position within mitochondrial genomes of eimeriid coccidia. Int J Parasitol.

[50] Katoh K, Standley DM (2013). MAFFT multiple sequence alignment software version 7: improvements in performance and usability. Mol Biol Evol.

[51] Huelsenbeck JP, Ronquist F (2001). MRBAYES: Bayesian inference of phylogenetic trees. Bioinformatics.

[52] Darriba D, Taboada GL, Doallo R, Posada D (2012). jModelTest 2: more models, new heuristics and parallel computing. Nat Methods.

[53] FaBox (1.41) - an online fasta sequence toolbox: Aarhus University; 2013 [cited 2018 25 April]. Available from: http://users-birc.au.dk/biopv/php/fabox/.

[54] Bandelt HJ, Forster P, Rohl A (1999). Median-joining networks for inferring intraspecific phylogenies. Mol Biol Evol.

