# Supplementary material for: The linear mitochondrial genome of the quarantine chytrid Synchytrium endobioticum; insights into the evolution and recent history of an obligate biotrophic plant pathogen
Source: BMC Evol Biol. 2018 Sep 10;18:136. doi: 10.1186/s12862-018-1246-6 (PMC6131824; doi:10.1186/s12862-018-1246-6)
Supplement: Supplementary file 1 — Figure S1. PacBio read mapping to the S. endobioticum mtDNA. Figure S2. Dotplot internal repeat structure TIRs Figure S3. Dotplot repeat in AT-rich region containing dpoB gene Figure S4. Interspecies comparison of organisation and orientation of mitochondrial genes, tRNAs and rRNAs Figure S5. Haplotype network based on CDS of mtDNA genes S. endobioticum Figure S6. Within-strain diversity in haplotype network based on polymorphic sites of the S. endobioticum mtDNA Figure S7. Synchytrium taraxaci Taqman assay Figure S8. TdT-tailing controls. (DOCX 2313 kb) [file 12862_2018_1246_MOESM1_ESM.docx]

**The linear mitochondrial genome of the quarantine chytrid *Synchytrium endobioticum*; insights into the evolution and recent history of an obligate biotrophic plant pathogen**

B.T.L.H. van de Vossenberg^1,2^, B. Brankovics^3^, H.D.T. Nguyen^4^, M.P.E. van Gent-Pelzer^1^, D. Smith^5^, K. Dadej^4^, J. Przetakiewicz^6^, J. Kreuze^7^, M. Boerma^8^, G. van Leeuwen^2^, C.A. Lévesque^4^, T.A.J. van der Lee^1^

* Corresponding author: [b.t.l.h.vandevossenberg@nvwa.nl](mailto:b.t.l.h.vandevossenberg@nvwa.nl), 0031 317 496 911

1. Wageningen UR, Droevendaalsesteeg 1, 6708 PB, Wageningen, the Netherlands

2. Dutch National Plant Protection Organization, National Reference Centre, Geertjesweg 15, 6706EA, Wageningen, The Netherlands

3. Westerdijk Fungal Biodiversity Institute, Uppsalalaan 8, 3584 CT, Utrecht, Netherlands

4. Agriculture and Agri-Food Canada, 960 Carling Avenue, Ottawa, Canada

5. Canadian Food Inspection Agency, 93 Mount Edward Road, Charlottetown, Canada

6. Plant Breeding and Acclimatization Institute, National Research Institute, Radzikow, 05-870 Blonie, Poland

7. International Potato Centre, Avenida La Molina 1895, Lima, Peru

8. Hilbrands Laboratorium BV, Kampsweg 27, 9418 PD Wijster, the Netherlands

**Supplementary information**

**1. PacBio read mapping to the *S. endobioticum* mtDNA**

**2. Dotplot internal repeat structure TIRs**

**3. Dotplot repeat in AT-rich region containing *dpoB* gene**

**4. Interspecies comparison of organisation and orientation of mitochondrial genes, tRNAs and rRNAs**

**5. Haplotype network based on CDS of mtDNA genes *S. endobioticum***

**6. Within-isolate diversity in haplotype network based on polymorphic sites of the *S. endobioticum* mtDNA**

**7. *Synchytrium taraxaci* Taqman assay**

**8. TdT-tailing controls**

**1. PacBio read mapping to the *S. endobioticum* mtDNA**


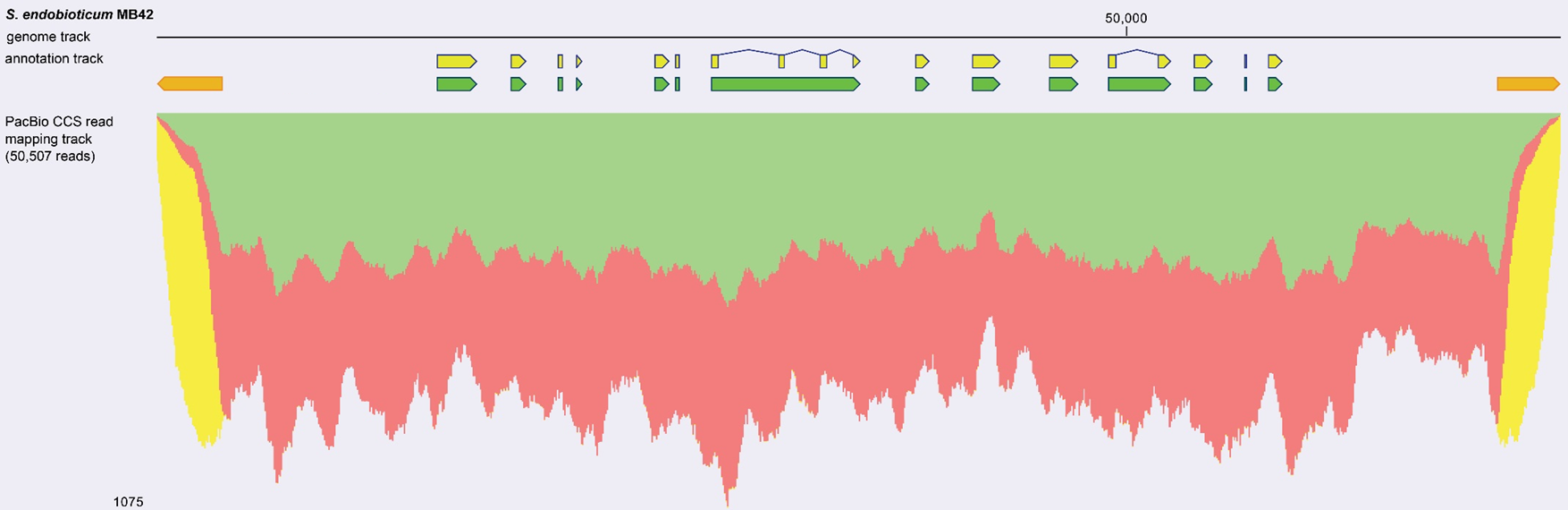


**SI Figure 1.** Mapping of 50,507 PacBio circular consensus reads to the 72,865 bp linear *S. endobioticum* mtDNA genome. Annotations for mitochondrial genes (green), CDS (yellow), and the Terminal Inverted Repeats (orange) are provided in the annotation track. Mapped reads (forward reads: green; reverse reads: red; unspecific match: yellow) are shown in the mapping track. Reads mapping to the terminal inverted repeats are shown in yellow since they can map equally well at both terminal ends of the assembly. PacBio reads map contiguously to the mtDNA template, and no reads exceed the 5’ or 3’ terminal end of the reference sequence.

**2. Dotplot internal repeat structure TIRs**


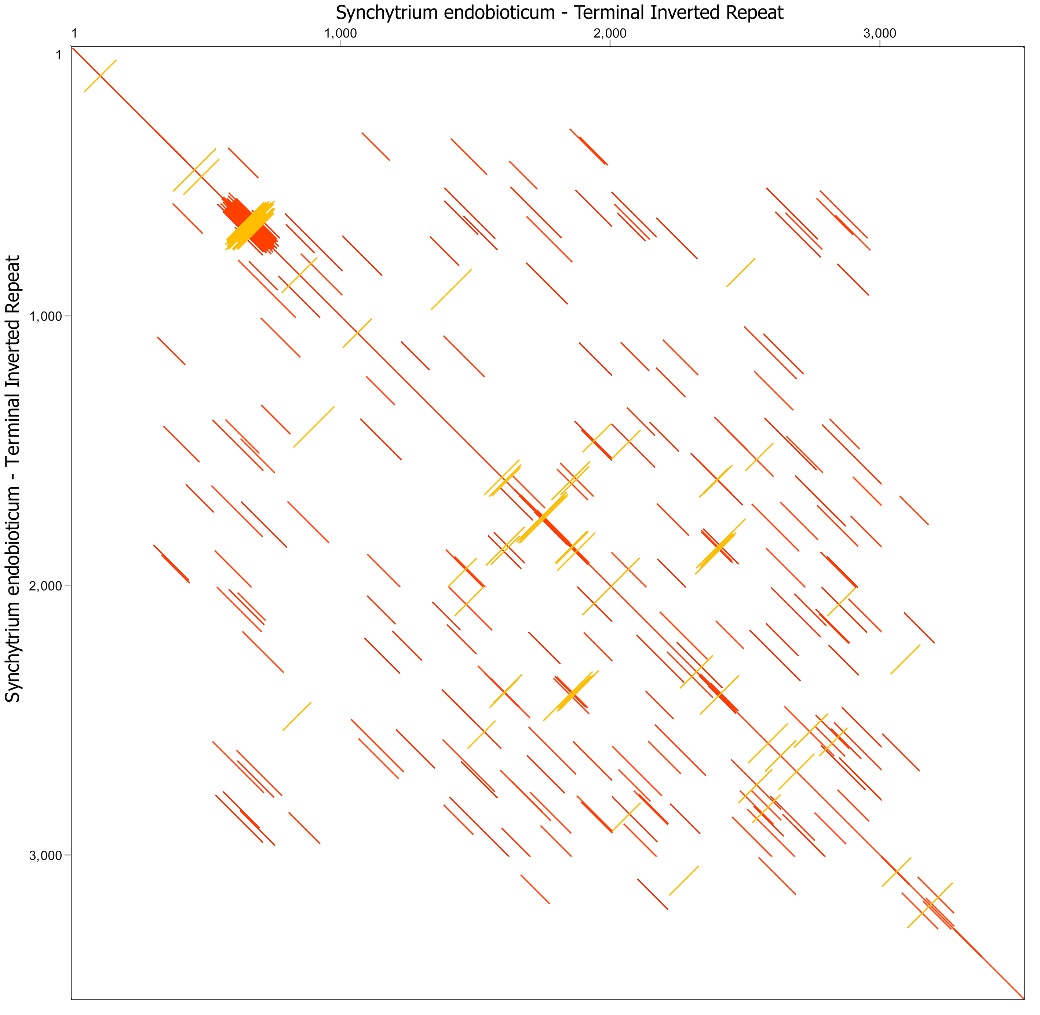


**SI Figure 2.** Dotplot (self) of the 3,529 bp Terminal Inverted Repeat (TIR) of the 3’ end of the linear *S. endobioticum* mtDNA. All positions from the TIR are compared with itself using the EDNAFULL (no ambiguous matches) substitution matrix using a window size of 100 bases and a similarity threshold of 50%. Similarities between a position from each sequence are plotted (in frame = red, reverse complement = orange).

**3. Dotplot repeat in AT-rich region containing *dpoB* gene**


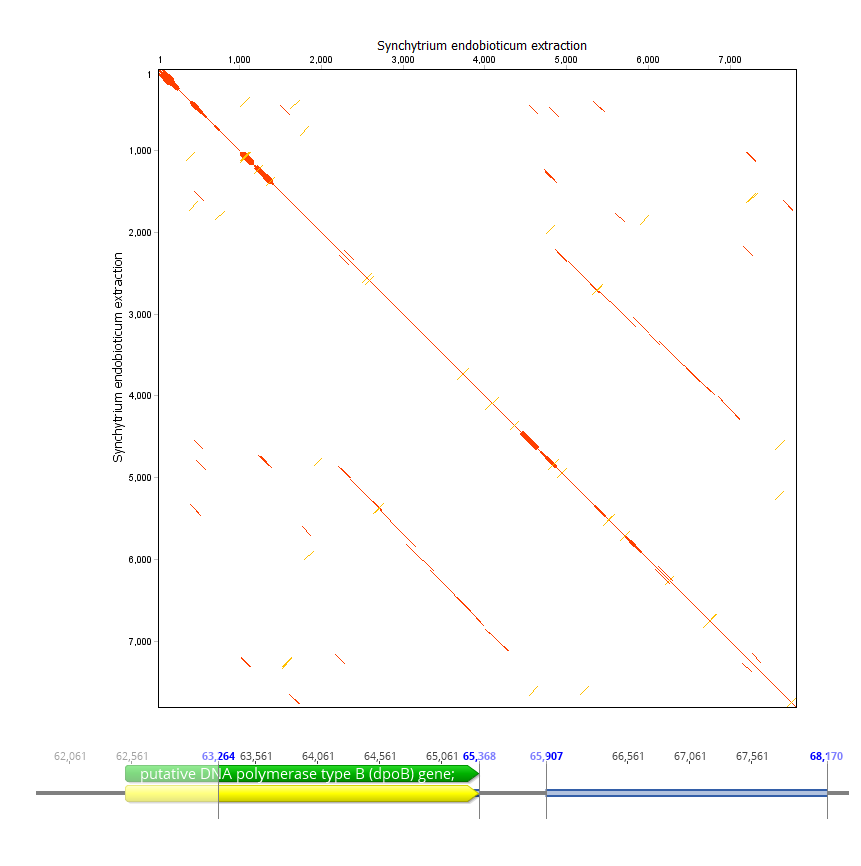


**SI Figure 3.** Dotplot (self) of the 7,524 bp A-T rich region of the linear *S. endobioticum* mtDNA containing the *dpoB* gene. All positions from the A-T rich region are compared with itself using the EDNAFULL (no ambiguous matches) substitution matrix using a window size of 100 bases and a similarity threshold of 50%. Similarities between a position from each sequence are plotted (in frame = red, reverse complement = orange). The *dpoB* gene and its repeated sequence (similarity 54.7%) are highlighted in an annotated sequence track.

**4. Interspecies comparison of organisation and orientation of mitochondrial genes, tRNAs and rRNAs**

**
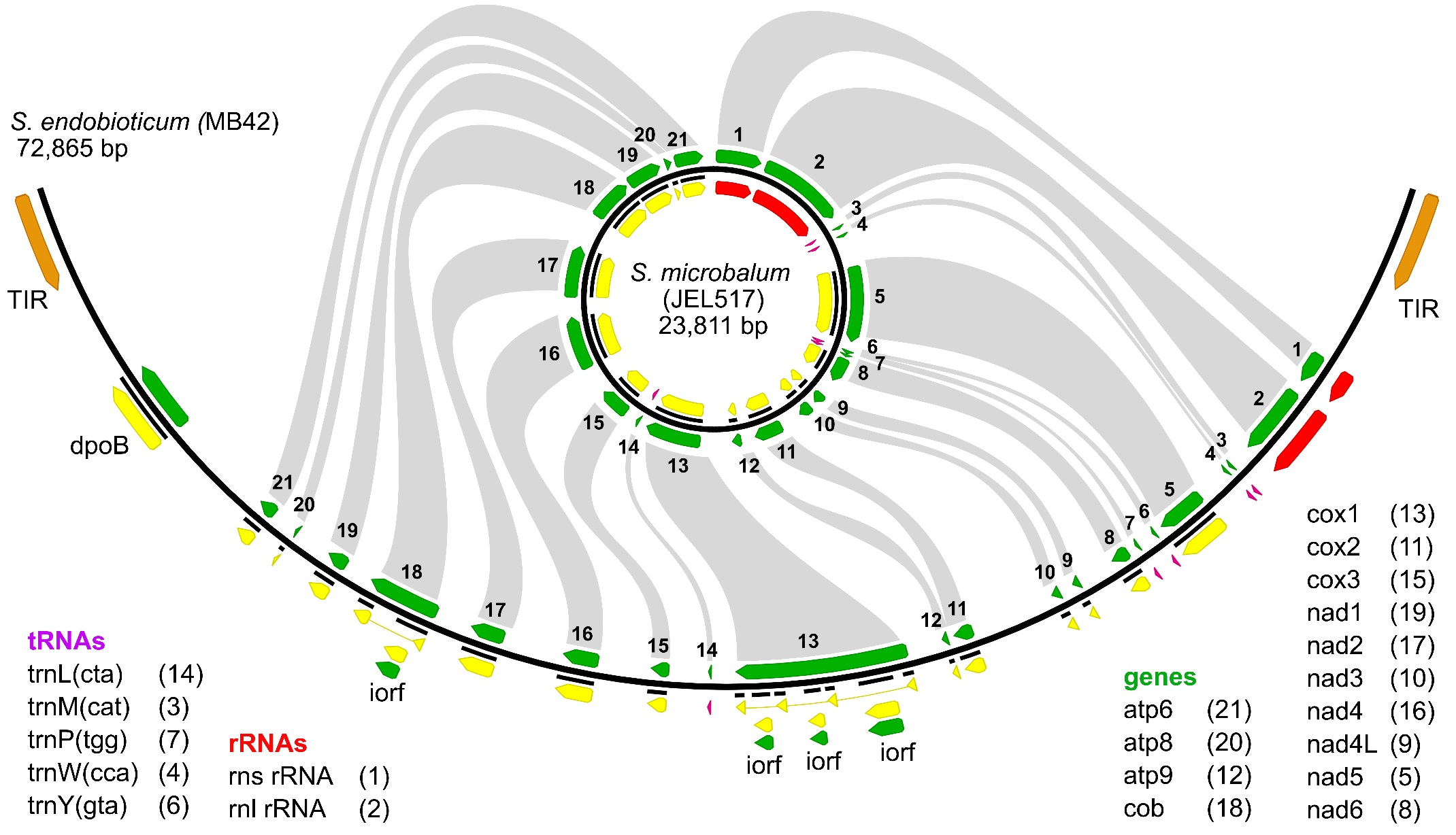
**

**SI Figure 4.** Comparison of the mitochondrial genomes of *S. endobioticum* MB42 and *S. microbalum* JEL517. Organisation and orientation of the mitochondrial genes, tRNAs and rRNAs is conserved between the 72,865 bp linear mtDNA of *S. endobioticum* and the circular mapping 23,811 bp mtDNA genome of *S. microbalum*. Genes are annotated in green, CDS in yellow, tRNAs in purple, rRNAs in red, Terminal Inverted Repeats (TIR) in orange. Grey links between the two mtDNA genomes indicate the orthologous genes, tRNAs and rRNAs. Intronic open reading frames on the *S. endobioticum* genome are indicated “iorf”, and the DNA polymerase B gene is indicated “dpoB”.

**5. Haplotype network based on polymorphisms in CDS of mtDNA genes**

Comparing 30 *S. endobioticum* isolates, 19 polymorphic sites were found in seven mitochondrial genes (*atp6*, *cob*, *cox1*, *nad1*, *nad4*, *nad5* and *nad6*). Three of the observed SNPs resulted in non-synonymous (dN) substitutions relative to the 1(D1) MB42 mtDNA: two in *atp6*, and one in *nad5*. In addition, the insertion of 6 bases resulted in the gain of two amino acids in *nad5* relative to the reference. Clustering of the informative sites using a Median Joining network resulted 10 different mitochondrial haplotypes (SI Fig. 5, SI Table 3). Both Dutch pathotype 1(D1) isolates together form haplotype 1, and the Polish pathotype 1(D1) isolate and the pathotype 6(O1) lab isolate obtained after two multiplications on cultivar Erika cluster with isolates from Germany and Belarus in haplotype 5. All Canadian 6(O1) isolates cluster together with a Dutch pathotype 2(G1) isolate in haplotype 3. Haplotype 2 contains several Polish isolates, a isolate from Ukraine and a Danish isolate with diverse pathotype identities. Haplotype group 7 contains the European pathotype 6(O1) isolates together with a German pathotype 2(G1) isolate. Pathotype 18(T1) isolates are restricted to haplotype group 8, and isolates of pathotype 8(F1) are found in haplotypes 8 and 9. The pathotype 2(G1) isolate from Germany is a singleton, as are the Peruvian and Russian isolates with unknown pathotype identify: haplotypes 4, 6 and 10 respectively.


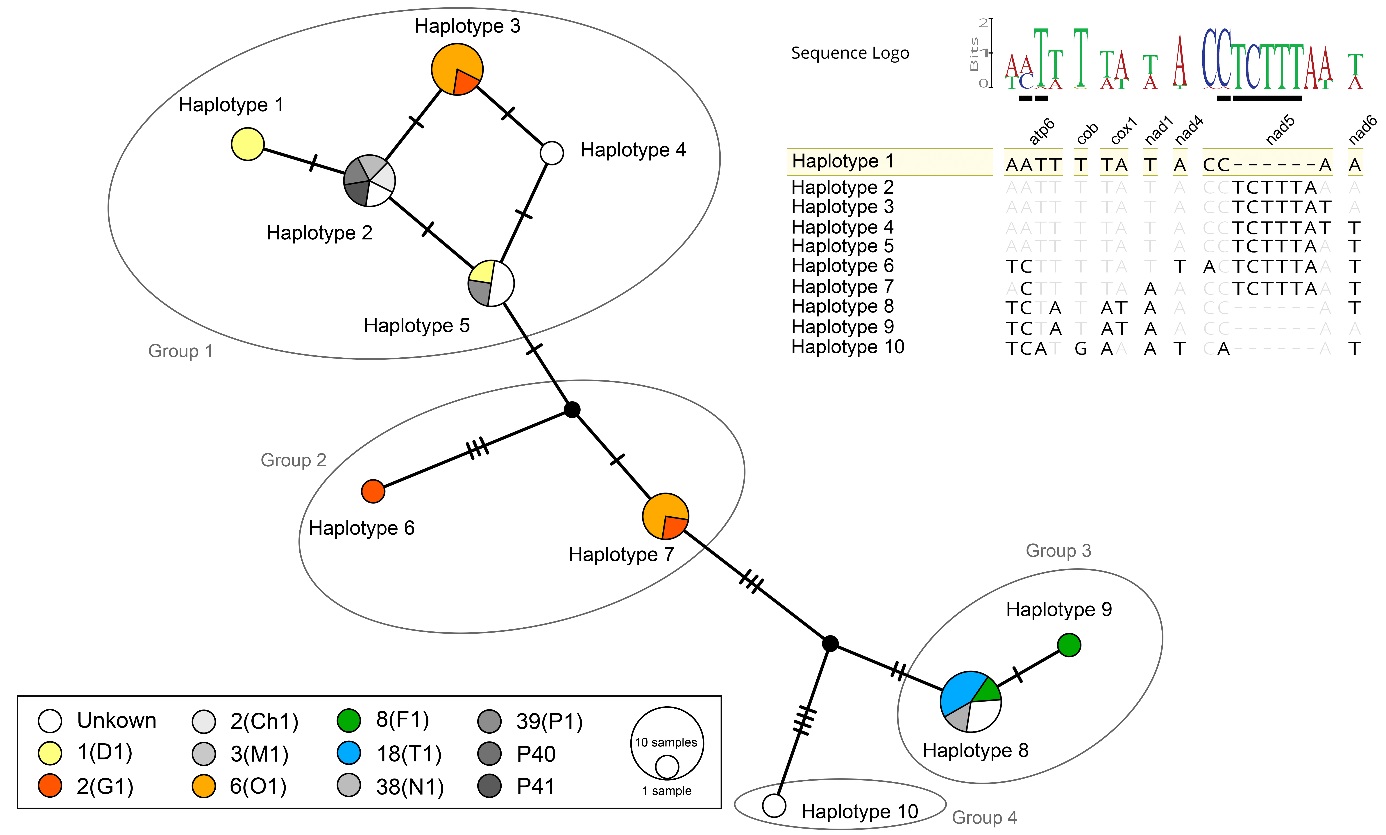


**SI Figure 5.** Median Joining haplotype network based on polymorphic sites in seven mitochondrial genes. Nodes in the network are coloured based on pathotype identity. Colours are used for pathotypes of major importance in Europe and Canada, whereas a greyscale is used for pathotypes of lesser importance (1). Black nodes represent hypothetical ancestors. Marks on the branches indicate the number of mutations. Sequences of the informative sites and their corresponding genes are provided as an alignment using the genotype of isolate MB42 (haplotype 1) as reference. Differences to the reference are highlighted. The sequence logo shows the incidence of the different bases in the set of 30 *S. endobioticum* isolates analysed. Underlined bases in the sequence logo represent nucleotide changes that have an effect on the amino acid sequence. Assignation of isolates to mtDNA haplotypes are provided in SI Table 3.

**6. Within-isolate diversity in haplotype network based on polymorphic sites of the *S. endobioticum* mtDNA**

**
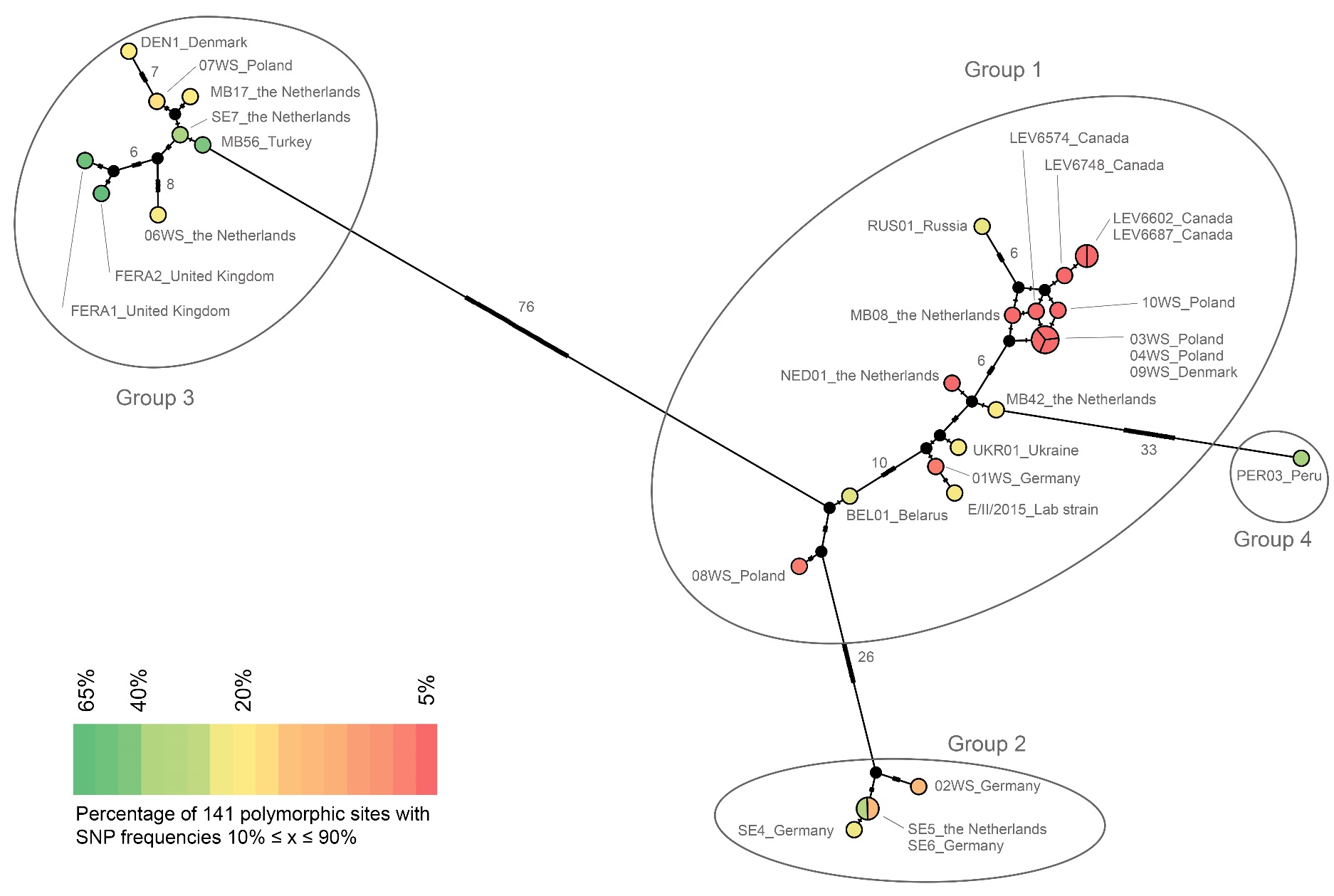
**

**SI Figure 6.** Median Joining haplotype network based on 141 polymorphic sites of the mtDNA. Nodes in the network are coloured based on the within-isolate diversity expressed as the percentage of polymorphic sites with intermediate (10% ≤ x SNP ≤ 90%) frequencies. Black nodes represent hypothetical ancestors. Isolates from group three and the Peruvian isolate (group four) show high within-isolate diversity while several isolates from group one are scarce in diversity.

**7. *Synchytrium taraxaci* Taqman assay**

Thirty-nine *Synchytrium* spp. sequences of the (partial) ribosomal DNA cassette (18S, ITS1, 5.8S, ITS2, and 28S) were downloaded from NCBI GenBank: *Synchytrium athyrii* (KF160863), *Synchytrium aureum* (KF160864), *Synchytrium australe* (KF160865), *Synchytrium collapsum* (KF160866), *Synchytrium cupulatum* (KF160867), *Synchytrium decipiens* (AY997094, DQ273819, DQ536475, KF160868), *Synchytrium endobioticum* (AJ784274, AY854021, JF795579, JF795580, KF160869, KF160870, KF160871, KF160872), *Synchytrium epilobii* (KF160873), *Synchytrium fulgens* (KF160874), *Synchytrium macrosporum* (AY997095, DQ273820, DQ322623, NG_017170, NG_027565), *Synchytrium minutum* (HQ324138, HQ324139), *Synchytrium papillatum* (JX093568, KF160875), *Synchytrium perforatum* (KF160876), *Synchytrium plantagineum* (KF160877), *Synchytrium puerariae* (EF053261, EF053262, EF053263), *Synchytrium* sp. CAL-2013 (KF160861, KF160862), *Synchytrium* sp. DAOM 240977 (HQ317546), *Synchytrium* sp. DUH0009364 (DQ536476), *Synchytrium stachydis* (KF160878), and *Synchytrium* *taraxaci* (KF160879). Alignments of ITS1, 5.8S and ITS2 allowed inclusion of the highest number of specimens in the analysis, and the ITS2 region was chosen for the primer and probe design (Fig. 1). *In-silico* analysis of primer and probe specificity showed that no non-specific amplification is to be expected from the species included in the design (Geneious R10, mismatches allowed = 3).


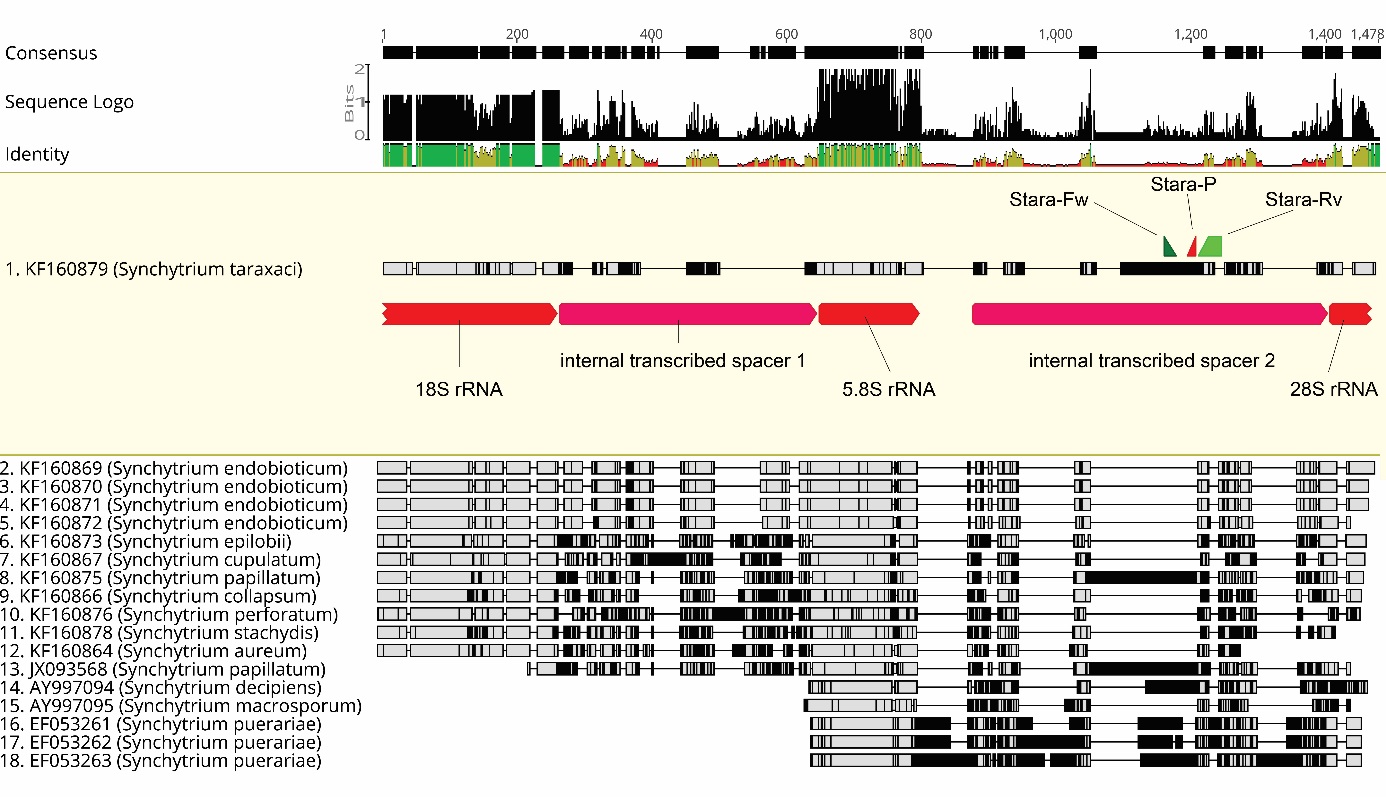


**SI Figure 7.** MAFFT alignment of 18 partial ribosomal cassette sequences covering 13 *Synchytrium* species: *S. aureum*, *S. collapsum*, *S. cupulatum* *S. decipiens*, *S. endobioticum*, *S. epilobii*, *S. macrosporum*, *S. papillatum*, *S. perforatum*, *S. plantagineum*, *S. puerariae*, *S. stachydis*, and *S. taraxaci*. Sequence KF160879 (*S. taraxaci*) serves as reference sequence and is displayed on top of the alignment, remaining sequences are sorted by length. Bases in alignment similar to the reference are shown in grey and differences to the reference are highlighted (black). Ribosomal RNA genes (red) and the internal transcribed spacers (purple) are annotated on the reference sequence, as well as the annealing sites for the *S. taraxaci* specific primers Stara-Fw (dark green), Stara-Rv (light green) and probe Stara-P (Red).

Real-time PCR reactions targeting the *S. taraxaci* ITS2 region were performed in a total volume of 30 µL containing 1x Premix Ex Taq (TaKaRa), 250 nM of each primer (Stara-fw: 5’-GTTGTTTGGACCTTTTGTTCCG-3’, Stara-rv: 5’GAGTGGAAGATCAAAGGCATCTC-3’), 83 nM of probe (Stara-p: 5’ FAM- CTGCATGACAGGACTGT -MGB 3’), and 3 µL genomic DNA . Real-time PCR reactions were carried out in an ABI PRISM 7500 Sequence detector (ThermoFisher) using the following cycling conditions: 2 min at 94 °C, 40 cycles of 15 sec at 95 °C and 1 min at 60 °C. A synthetic construct (IDT, United States of America) of the *S. taraxaci* ITS accession KF160879 was used as positive amplification control to monitor the amplification efficiency. Cq values obtained for *S. taraxaci* spores extracted from dandelion leaves ranged from 19.9 to 23.3. No false positive results were obtained when testing *S. endobioticum* or *S. microbalum* DNA.

**8. TdT-tailing controls**

Positive and negative controls were included to monitor the effectiveness of the TdT tailing. The TdT tailing control consists of a 3747 bp mtDNA insert cloned in a pUCIDT (IDT) high copy vector. The mitochondrial insert sequence contains the terminal inverted repeat (TIR) sequence, with modifications to allow synthesis, containing primer sites mtDNA_00007-fw and mtDNA00787-rv, the 5’ specific sequence with primer site mtDNA_03691-rv, and the 3’specific sequence with primer site mtDNA_69209-fw. After digestion with BsmBI (New England Biolabs), a linear 6041 bp fragment containing the mtDNA insert is obtained together with two smaller linear fragments (416 bp and 42 bp). In its circular form the plasmid acts as a negative control for the TdT-tailing reaction, and after digestion the linear plasmid fragments are used as positive control. Efficiency of digestion is monitored using primer pair M13F/mtDNA_00787-rv: without digestion an amplicon is expected with this primer pair, and after full digestion no amplicon is expected since the plasmid is cleaved between primer site M13F and mtDNA_00787-rv (Fig 8a). Primer M13F is not to be confused with primer M13FpolyG. The first anneals to a primer site on the circular TdT control, and forms a pair with primer mtDNA00787-rv: i.e. a control for digestion The latter anneals to the digested, linear fragment after TdT tailing (poly-C addition), and forms a pair with primer mtDNA00787-rv: i.e. a control for TdT tailing.


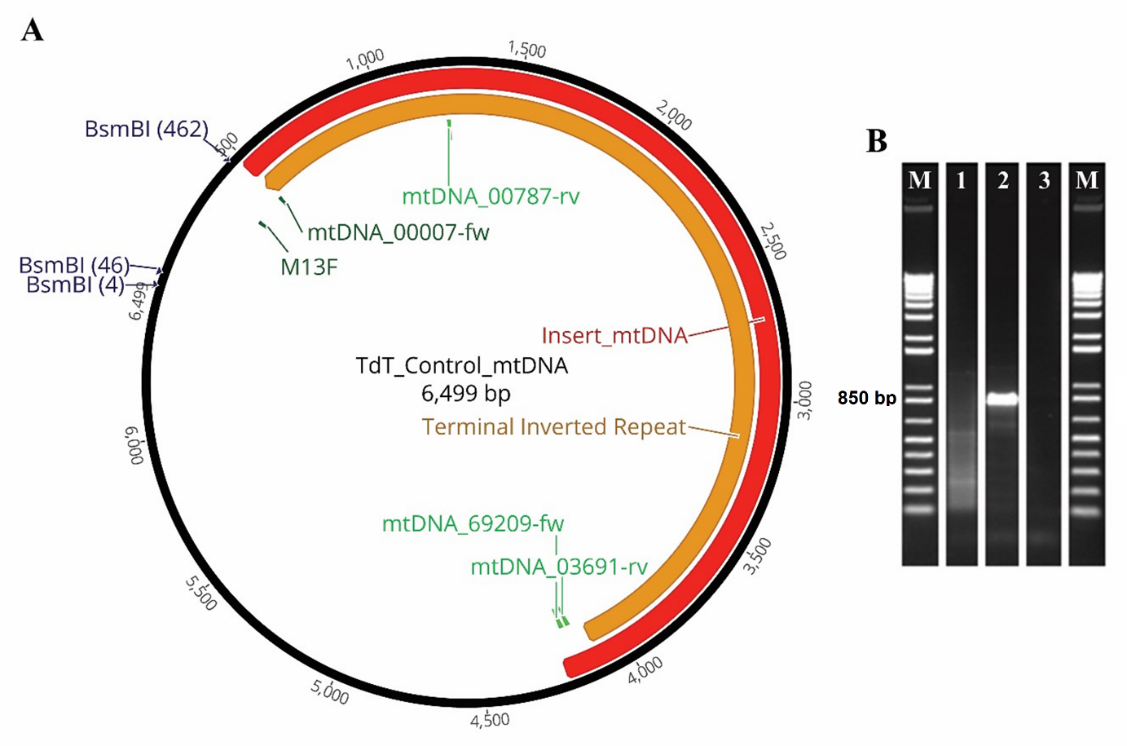


**SI Figure 8.** **A** Circular conformation of the TdT control annotated with the mtDNA insert sequence (red), the modified TIR (orange), forward orientated primers (dark green), reverse orientated sequences (light green), and BsmBI restriction sites. **B** Gel image of PCR reactions after TdT tailing using primer pair M13FpolyG x mtDNA_00787-rv. The following samples were tested: 1. undigested TdT control, 2. BsmBI digested TdT control, 3. molecular grade water. Estimation of amplicon size was done based on the Invitrogen 1kb plus maker (M).

**References**

1. Baayen RP, Cochius G, Hendriks H, Meffert JP, Bakker J, Bekker M, et al. History of potato wart disease in Europe – a proposal for harmonisation in defining pathotypes. European Journal of Plant Pathology. 2006;116(1):21-31.
